# Exosomal circRNA RHOT1 promotes breast cancer progression by targeting miR-204-5p/ PRMT5 axis

**DOI:** 10.1186/s12935-023-03111-5

**Published:** 2023-11-03

**Authors:** Weihua Jiang, YinPing Yu, Jianghua Ou, Yongtao Li, Ning Zhu

**Affiliations:** 1 Department of Breast Surgery, The Affiliated Tumor Hospital of Xinjiang Medical University, Urumqi, 830000 Xinjiang China; 2 Medical School, Hunan University of Medicine, No. 492, Jinxi South Road, Hecheng District, Huaihua, 418000 Hunan China

**Keywords:** Breast Cancer, Exosome, CircRHOT1, MiR-204-5p, PRMT5

## Abstract

**Background:**

Circular RNA RHOT1 (circRHOT1) plays crucial roles in tumorigenesis by competing with microRNAs. It is largely abundant in tumor cell-derived exosomes. Meanwhile, cancer-derived exosomes participate in diverse biological processes. However, the expression patterns and functions of exosomal circRHOT1 in breast cancer remain unknown. This study is aimed to investigate and elucidate the exosomal circRHOT1/miR-204-5p/PRMT5 axis in breast cancer.

**Methods:**

The exosomes derived from serum samples of breast cancer patients and breast cancer cell lines were characterized using transmission electron microscopy and Western blot. MTT, colony formation, wound healing, and transwell assays were utilized to analyze cell proliferation, migration, and invasion of breast cancer cells. Flow cytometry was used for apoptosis analysis. The bioinformatics method was employed to screen differentially expressed novel circRNAs and predict the microRNA targets of circRHOT1. Dual-luciferase reporter gene assays were performed to verify their direct interaction. Finally, Xenograft experiments were used to investigate the effect of exosomal circRHOT1 on tumor growth in vivo.

**Results:**

CircRHOT1 exhibited significantly high expression in exosomes derived from the serum of breast cancer patients and breast cancer cell lines, which suggested its potential diagnostic value. Breast cancer-derived exosomes promoted the cell proliferation, migration, invasion, and epithelial-mesenchymal transition of breast cancer cells while inhibiting apoptosis. However, exosomes with downregulated circRHOT1 inhibited the growth of co-cultured cells. Mechanistically, circRHOT1 acted as a sponge of miR-204-5p and promoted protein arginine methyltransferase 5 (PRMT5) expression. Moreover, miR-204-5p inhibitor and pcPRMT5 could reverse the tumor suppressive effects mediated by circRHOT1-knockdown. Furthermore, treatment with exosomes derived from breast cancer cells with circRHOT1 knockdown attenuated tumor growth in tumor-bearing nude mice, which was accompanied by a reduction in PRMT5 expression and an enhancement of miR-204-5p expression.

**Conclusion:**

The exosomal circRHOT1 may promote breast cancer progression by regulating the miR-204-5p/PRMT5 axis. The current study strengthens the role of circRHOT1, miR-204-5p, and PRMT5 in breast cancer development and provides a potential treatment strategy for breast cancer.

**Supplementary Information:**

The online version contains supplementary material available at 10.1186/s12935-023-03111-5.

## Background

Breast cancer is one of the most prevalent diseases among women with a high mortality rate. In 2020, there were over 2 million new cases of breast cancer, comprising 11.7% of all new cancer cases. Additionally, breast cancer caused over 68 thousand deaths, accounting for 6.9% of all cancer-related deaths [1]. With advances in diagnosis and treatments, the prognosis of breast cancer has dramatically improved [2]. However, metastatic breast cancer remains a major cause of cancer-related deaths and poses a significant challenge to effective treatment [3]. Therefore, a more comprehensive understanding of the mechanism of progression and development of breast cancer is important for identifying new therapeutic targets.

Exosomes, a subset of extracellular vesicles (EVs) with sizes ranging from 30 to 150 nm, have emerged as key players in cancer treatment [4, 5]. Exosomes contain various active substances, including circular RNA (circRNAs) [6], microRNA (miRNA) [7], and proteins [6, 8, 9]. These components have been observed to be released into the extracellular space through exosomes, facilitating the transport of functional molecules to nearby or distant cells [10–12]. Therefore, exosomes play crucial roles in regulating the growth, metastasis, and angiogenesis of various cancers [6, 13], including breast cancer [14–16], colorectal cancer [17], and gastric cancer [18]. Additionally, exosomes can be used as prognostic biomarkers for cancer patients [19].

CircRNAs are single-stranded non-coding RNAs that are covalently bound at the 3′ and 5′ ends after reverse splicing [20]. They play a role in regulating gene expression and modulating protein function by acting as miRNA sponges or combining with proteins [21]. CircRNAs are abundant in exosomes and exhibit remarkable stability. Hence, they have been implicated in diverse cancer-related processes [22, 23], including those of breast cancer [24, 25]. Recently, there has been growing interest in the role of circRNAs as intercellular messengers through their transfer via EVs. CircRNAs, with their unique structure and stability, can be packaged and released within various types of EVs, such as exosomes and microvesicles. It has been demonstrated that circRNAs can regulate the generation and release of EVs [26], thereby influencing the physiological processes of recipient cells. Circular RNA RHOT1 (circRHOT1), as a newly identified circRNA, is derived from the RHOT1 gene. In hepatocellular carcinoma, circHOT1 functions as an oncogene by upregulating the expression of NR2F6 and recruiting TIP60 [27]. A previous study has demonstrated that circHOT1 functions as a miRNA sponge in pancreatic cancer [28]. However, the association between circRHOT1 and exosomes in breast cancer remains unknown.

The miRNAs are a class of endogenous 22-25nt non-coding RNA molecules that act as key regulators in posttranscriptional gene regulation [29]. Hong et al. suggested that miR-204-5p had the potential to inhibit breast cancer metastasis and immune cell reprogramming by regulating the PI3K/Akt signaling pathway [30]. Additionally, circPVT1 promotes the invasion and epithelial-mesenchymal transition (EMT) of breast cancer cells by serving as a competing endogenous RNA for miR-204-5p [31]. However, the relationship between circRHOT1 and miR-204-5p has yet to be investigated.

Protein arginine methyltransferase 5 (PRMT5) is a member of the arginine methyltransferase family [32]. It is widely present in the cytoplasm and nucleus. Moreover, it plays a crucial role in catalyzing the symmetric methylation of multiple substrates, including histones and non-substrates, thereby affecting multiple target genes and signaling pathways and exerting multiple biological functions [33, 34]. It has been shown that PRMT5 regulates cell proliferation, differentiation, and apoptosis. Its expression is upregulated in various human malignancies. For instance, Yao et al. demonstrated that PRMT5 aggravated prostate cancer progression by inhibiting the transcription of CAMK2N1, and was modulated by the circSPON2/miR-331-3p axis [35]. However, the relationship between circRHOT1 and PRMT5 in breast cancer remains unexplored.

In this study, we presented novel findings regarding the presence of circRHOT1 in exosomes derived from breast cancer cells. Furthermore, we investigated the function and potential mechanism of circRHOT1 in breast cancer progression. Our results demonstrate that circRHOT1 could promote breast cancer development by targeting miR-204-5p/PRMT5 axis. This study contributes to the understanding of breast cancer pathogenesis and provides potential therapeutic targets for the treatment of this disease.

## Methods

### Ethics

This study was carried out following the World Medical Association’s Declaration of Helsinki and was approved by the Research Ethics Committee in The Affiliated Tumor Hospital of Xinjiang Medical University. Written informed consent was obtained from all participants.

### Study participants

Patients diagnosed with primary breast cancer (n = 10) were enrolled from the Affiliated Tumor Hospital of Xinjiang Medical University. Inclusion criteria: (1) Patients with breast cancer confirmed by routine pathology after surgical resection; (2) patients who were treatment naïve before blood collection. Exclusion criteria: (1) patients with a previous history of malignancy; (2) patients complicated with other tumors; (3) patients with incomplete clinical data. For control, 10 healthy individuals who received physical examination during the same period were also enrolled. Blood samples were collected from each participant.

### Cell culture and transfection

MCF-10 A and MCF-7 cell lines were purchased from the American Type Culture Collection. The MCF-7 cells were cultured in DMEM medium (Gibco, USA) supplemented with 100 µg/mL streptomycin, 100 U/mL penicillin, and 10% exosome-free fetal bovine serum at 37 °C, 5% CO2. Additionally, MCF10A was cultured in DMEM/F12 medium supplemented with 10% horse serum, 20 ng/mL epidermal growth factor, 10 µg/mL insulin, 100 ng/ml cholera toxin, and 50 µg/mL hydrocortisone.

The plasmids carrying circRHOT1 siRNA (5’-AGACAAAGACAGCAGGTTCCT-3’) (si-circRHOT1) and the corresponding negative control siRNA (si-NC), pcDNA3.1-circRHOT1 overexpression plasmid, pcDNA3.1-PRMT5 overexpression plasmid, miR-204-5p mimics, miR-204-5p inhibitor, and the corresponding control (Anhui General Biology Company, China) were synthesized. The cells were transfected with these plasmids using Lipofectamine 2000 reagent (Thermo Fisher, Scientific, USA).

### Exosome isolation

The exosomes were extracted as previously described [36, 37]. Briefly, after transfection, the cell culture supernatants were collected and centrifuged at 4 °C. Then, the centrifugation supernatant was subjected to centrifugation at 10,000 Xg for 20 min. The resulting supernatant was further centrifuged at 100,000 Xg at 4 °C for 70 min. After that, the supernatants were filtered with a 0.22 μm filter, and the filtrate was centrifuged at 100,000 Xg for 1 h. The resulting precipitate was exosomes. Exosomes from serum were isolated by exoRNeasy Serum/Plasma Maxi Kit (Qiagen, Hilden, Germany). All exosomes were resuspended in PBS and stored at − 80 °C or used immediately for analysis.

### Transmission electron microscopy

The isolated exosomes were fixed with 1% glutaraldehyde for 20 min. Exosomes (10 µL) were then applied to the copper mesh of the electron microscope and the liquid was absorbed with filter paper after 15 min. The samples were negatively stained with 10 µL of 2% phosphotungstic acid at 37 °C for 2 min. Finally, the morphology was observed under the transmission electron microscope (Thermo Fisher, Waltham MA, USA).

### Co-culture with exosomes

MCF-7 cells (1 × 10^5^) were seeded and incubated with 20 µg/mL of exosomes. Cells were collected for subsequent experiments after 48 h incubation.

### MTT assay

Cells were seeded in 96 well plates (10^3^ cells/well) and incubated with or without inhibitor. After 48 h, the cells were incubated with 20 µL of MTT (M1020, Solarbio, Beijing, China) for 4 h at 37 °C. After adding 200 µL DMSO, the absorbance at 570 nm was measured to determine the number of cells.

### Flow cytometry

After different transfection, cells were collected and fixed in ice-cold 70% ethanol overnight at − 20 °C. Subsequently, the fixed cells were incubated with 100 µL of propidium iodide (50 µg/mL) for 30 min at 4 °C in the dark. The cell cycle was analyzed using a Cytomics FC 500 instrument (Beckman Coulter) equipped with CXP software and at least 10,000 cells were analyzed. To analyze apoptosis, cells were stained with Annexin V-FITC/ propidium iodide (Solarbio, Beijing, China), followed by the examination of apoptotic cells using flow cytometry (BD Biosciences, CA, USA).

### Colony formation assay

MCF-7 cells were seeded into 6-well plates (1000 cells/well). After a 2-week incubation period, the cells were collected, fixed in methanol for 30 min, and subsequently stained with 1% crystal violet. The number of colonies was calculated.

### Wound healing assay

MCF-10 A/ MCF-7 cells were plated into the 12-well plates and incubated overnight. Upon reaching 100% confluence, straight scratch lines were created using a 20 mL pipette tip. The cells were subsequently washed and cultured in a serum-free medium. The width of the wound was captured and measured at 0 and 24 h under a microscope. The wound healing percentage was calculated by the formula of (0 h scratch width – 24 h culture width) / 0 h scratch width× 100%.

### Transwell assays

Transwell chambers, which were pre-coated with Matrigel (RIRP12R48, Millipore, USA), were utilized to detect cell invasion of MCF-7/MCF-10 A. Briefly, 1 × 10^5^ cells were added to the upper chambers of the transwell plate (Corning, USA), while the lower chambers were supplemented with 500 µL of complete medium. After incubation at 37 °C for 24 h, the cells on the bottom of the upper chambers were fixed with 4% formaldehyde for 30 min and then stained with 0.1% crystal violet solution for 30 min. The images were then collected under five different fields of each sample. The number of invasive cells was counted by Image J.

### Bioinformatics analysis and target gene prediction

The GSE101124 dataset (https://www.ncbi.nlm.nih.gov/geo/geo2r/?acc=GSE101124) of the GEO database (https://www.ncbi.nlm.nih.gov/) was used to screen differentially expressed novel circRNAs. The screening criteria are log2FC > = 1 and P value < 0.05. For data preprocessing, we utilized R language, a widely used bioinformatics tool, to ensure data accuracy and reliability. Subsequently, we employed the “heatmap.2” function in R language to generate a circRNA expression heatmap. Additionally, the miRNAs and target genes directly associated with circ-RHOT1 were predicted by using the online prediction software circular RNA Interactome (https://circinteractome.nia.nih.gov/index.htm) and TargetScanHuman 7.2 (http://www.targetscan.org/vert_72/).

### Dual-luciferase reporter gene assay

The dual-luciferase reporter gene assays were performed as described previously [28]. Briefly, the MCF-7 and MCF-10 A cells were transfected with the miR-204-5p mimic, negative control for mimic (miR-NC), pmirGLO-circRHOT1 wild type, pmirGLO-circRHOT1 mutant, pmirGLOPRMT5 wild type, and pmirGLO-PRMT5 mutant by using Lipofectamine 2000 (Invitrogen, USA). After 48 h, the luciferase activities were analyzed with the Luciferase Assay System (Promega, WI, USA), in which Renilla was applied as a normalized control.

### Real-time quantitative PCR

Total RNA was extracted using Trizol reagent (Life Technologies, NY, USA), and was reverse transcribed using the RevertAid First Strand cDNA Synthesis Kit (EP0743, Takara, China). The relative miRNA and mRNA levels were examined using 1 × SYBR Green PCR master mix (Thermo Fisher, Waltham, MA, USA). The conditions were 95 °C for 15s, 58 °C for 30s, and 72 °C for 30s, with a total of 40 cycles. The internal control for miRNA and mRNA/circRNA was U6 and GAPDH, respectively. Before detecting circ-RHOT1 by quantitative real-time PCR, total RNA was subjected to RNase R (Thermo, USA) treatment at 37 °C for 20 min. Quantitative miRNA and mRNA levels were determined by triplicate independent experiments. The primer sequences are as follows:

GAPDH forward primer: 5’-CGGATTTGGTCGTATTGGG-3’.

GAPDH reverse primer: 5’-CTGGAAGATGGTGATGGGATT-3.

circRHOT1 forward primer: 5′-ATCACCATTCCAGCTGATGT-3′.

circRHOT1 reverse primer: 5′-TGCTGTCTTTGTCTGTTCTTTC-3′.

U6 forward primer: 5′-CTCGCTTCGGCAGCACA-3′.

U6 reverse primer: 5′-TGGTGTCGTGGAGTCG-3′.

miR-204-5p forward primer: 5′-GCTACAGTCTTTCTTCATGTG-3′.

miR-204-5p reverse primer: 5′-CCAGTGATGACAATTGAACG-3′.

### Western blot analysis

The total proteins were extracted from cells and their concentration was determined using the Pierce™ BCA protein Assay kit (Thermo Fisher). Proteins were separated by 10% SDS–PAGE and transferred to polyvinylidene fluoride membranes. The membranes were then blocked with 5% skim milk at 37 °C for 1 h. Subsequently, the membranes were incubated with the primary antibodies at 4 °C overnight, followed by incubation with the secondary antibody at 37 °C for 1 h. The target proteins were visualized using Enhanced chemiluminescence Western blotting detection reagents (Thermo Fisher). The antibodies were listed as follows: E-cadherin (Abcam, ab40772), N-cadherin (Abcam, ab18203), vimentin (Abcam, ab137321), GADPH (Abcam, ab8245), CD9 (Abcam, ab92726), and CD81 (Abcam, sc-9158).

### Construction of tumor-bearing nude mice

The animal experiments were approved by the Animal Experimentation Ethics Committee of the Affiliated Tumor Hospital of Xinjiang Medical University (approval no. K-2,021,046). All experiments were performed following relevant guidelines and regulations and followed the recommendations in the ARRIVAL guidelines. Male BALB/c nude mice (5 weeks old; n = 10) were selected and randomly divided into two groups. Every 3 days, nude mice were injected subcutaneously with exosomes derived from MCF-7 cells transfected with si-circRHOT1 or si-NC. The mice were monitored for 30 days. The body weight and tumor volumes (volume=(length×width^2^)/2) were measured every 3 days. At the end of the experiment, the mice were sacrificed and the tumors were dissected and weighed. Finally, the tumor tissues were analyzed by western blot and immunohistochemistry.

### Immunohistochemistry

Tissues were fixed in 4% formaldehyde, embedded in paraffin, and cut into 3-µm sections. Sections underwent dewaxing, re-hydration, antigen retrieval, and blocking, and then were incubated with primary antibodies overnight at 4 °C. After washing, the sections were incubated with HRP-conjugated secondary antibody (CST) for 15 min at 37 °C, washed three times with PBST, and then stained with DAB and hematoxylin. Next, sections were dehydrated and mounted with coverslips. The sections were scanned on Panoramic slide scanner II (3D HISTECH, Budapest, Hungary).

### Statistical analysis

Each experiment was repeated three times. All graphs were generated, and all statistical analyses were performed using GraphPad Prism 8.0. Differences between the two groups of data were tested by student’s t-test. One-way ANOVA was used for the analysis among multiple groups, with Bonferroni as a post hoc test. The diagnostic value of exosomal circRHOT1 in breast cancer was evaluated by the ROC (receiver operating characteristic) curve. Pearson’s correlation analysis evaluated the correlation between circ-RHOT1 and miR-204-5p. All data are expressed as mean ± standard deviation. P < 0.05 indicated significant differences.

## Results

### Serum exosomal circRHOT1 is relatively highly expressed in breast cancer and has a good diagnostic value

**Fig. 1:**
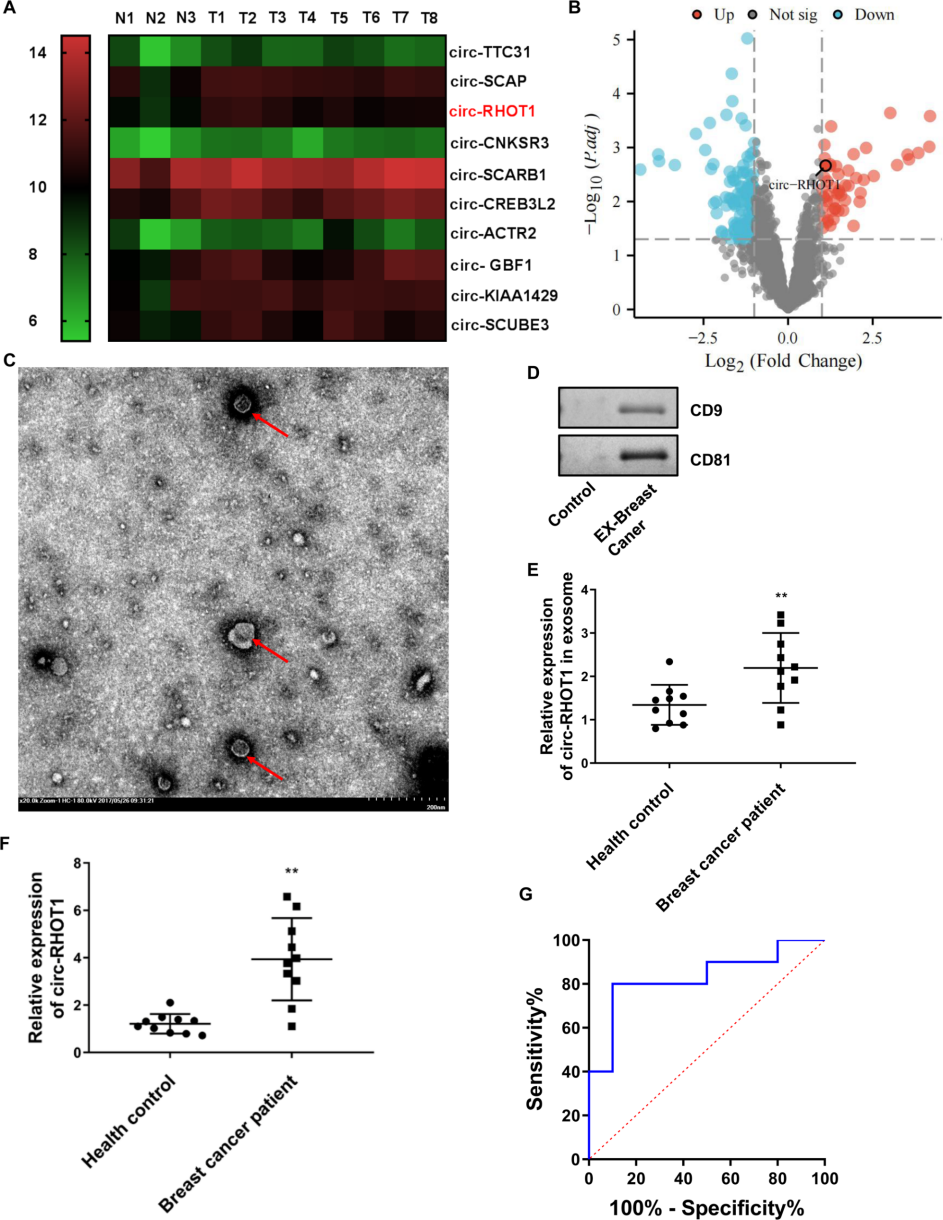
Serum exosomal circRHOT1 is relatively highly expressed in breast cancer and has a good diagnostic value **A**, Heatmap analysis of the circRNA expression profile in breast cancer (patients from the GSE101124 dataset). **B**, Volcano plot showing the circRNA expression profile in breast cancer (patients from the GSE101124 dataset). **C**, Representative images of exosomes isolated from breast cancer patients under the transmission electron microscope. (The arrows indicate the exosomes with a double-layer membrane). **D**, The expressions of exosome markers. The control group represented the residual cells after exosome isolation; while the EX-Breast Cancer group represented exosomes isolated from the serum of breast cancer. **E**, Expression of exosomal circRHOT1 in the serum of healthy control and breast cancer patients. The relative expression of circ-RHOT1 was normalized to GAPDH. **F**, Expression of circRHOT1 in tumor tissues of healthy control and breast cancer patients. **G**, The diagnostic value of exosomal circRHOT1 in breast cancer (AUC = 0.8300, *P*＜0.01). Compared with healthy control, ** p < 0.01

To identify crucial circRNA that may be involved in breast cancer progression, we analyzed the circRNA expression profiles in breast cancer using the GEO database (GSE101124). Our findings, as depicted in Fig. 1A and B, revealed that circRHOT1 exhibited significant upregulation in breast cancer tissue. Previous studies have demonstrated the increased expression of circRHOT1 in various cancers [27, 38]. Additionally, it has been demonstrated that circRHOT1 was presented in the exosome and had high clinical diagnostic values [39]. In this study, we examined the presence of circRHOT1 in serum exosomes derived from breast cancer patients. The serum exosome samples were collected from 10 breast cancer patients and 10 healthy controls. We performed transmission electron microscopy analysis of exosome morphology and Western blot analysis of protein biomarkers. The results indicated that the exosomes exhibited a cup-shaped or concave hemispherical morphology, with approximately 80–100 nm in diameter and a double-layer membrane (Fig. 1C). Moreover, the exosomes were found to be positive for CD9 and CD81 (Fig. 1D). Subsequently, circRHOT1 expression in serum exosomes and tumor tissues from breast cancer patients and healthy controls was investigated using quantitative real-time PCR. The analysis revealed a statistically significant increase in circRHOT1 levels in serum exosomes (Fig. 1E) and tumors (Fig. 1F) from breast cancer patients compared to those from healthy controls. Additionally, the diagnostic potential of exosomal circRHOT1 in breast cancer was evaluated using ROC analysis, The area under the curve of exosomal circRHOT1 was 0.8300 (*P* < 0.01) (Fig. 1G). This suggests that exosomal circRHOT1 can effectively distinguish between normal individuals and breast cancer patients.

### Breast cancer-derived exosomes promote breast cancer cell proliferation, wound healing, invasion, and EMT in vitro

**Fig. 2:**
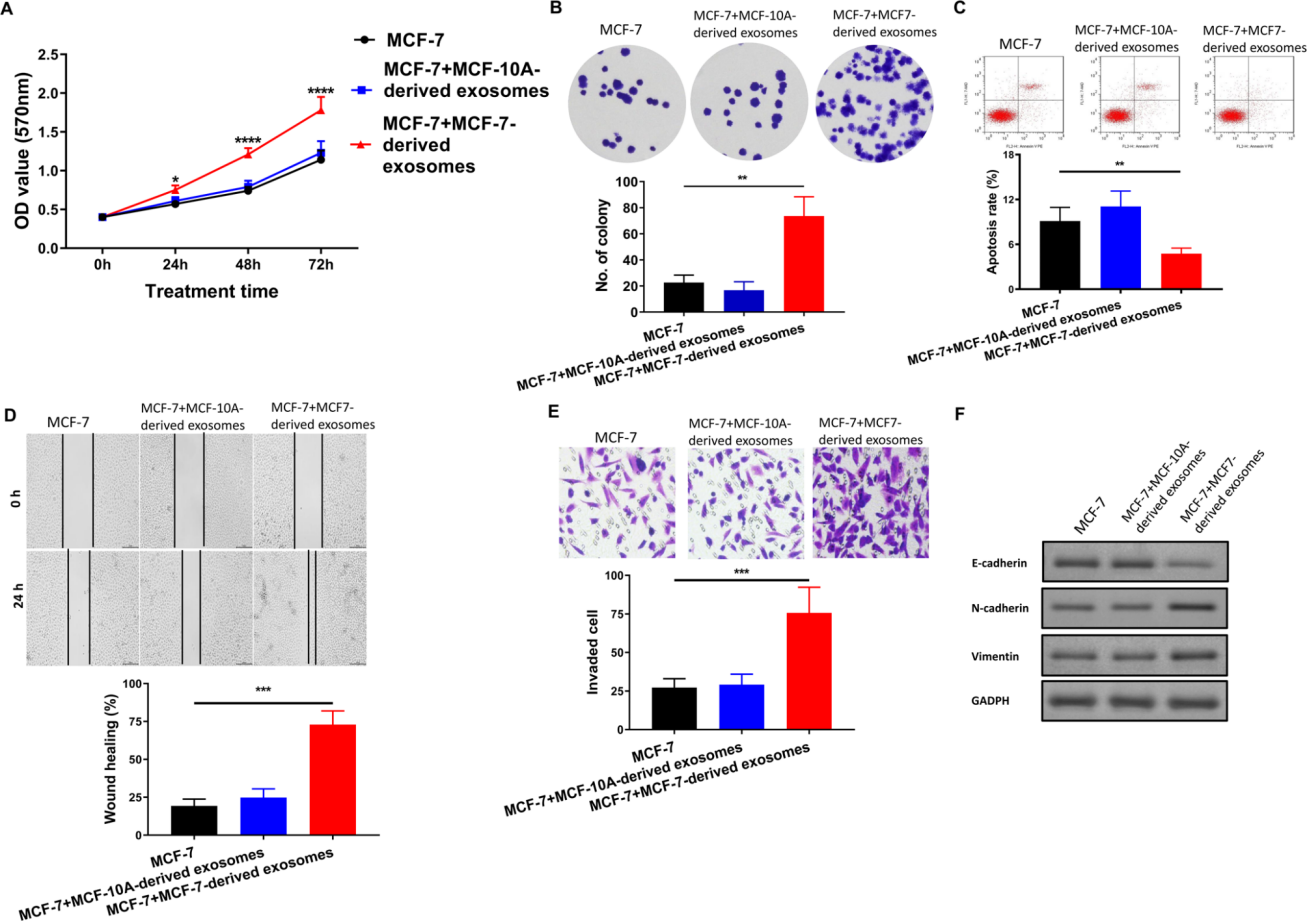
Exosomes derived from MCF-7 promote the proliferation, migration, and invasion of MCF-7. **A**, Cell proliferation assessed by MTT assay. **B**, Colony formation. **C**, Flow cytometry analysis of apoptosis. **D**, Wound healing assay results. **E**, Transwell assay of invasion. **F**, Western blot analysis of E-cadherin, N-cadherin, and vimentin expression. Compared with the MCF-7 and MCF-7 + MCF-10 A-derived exosomes, ** p < 0.01, *** p < 0.001

We isolated exosomes from the culture medium of MCF-10 A and MCF-7, respectively, and co-cultured them with MCF-7 cells to explore whether breast cancer-derived exosomes can affect the proliferation, invasion, and migration of breast cancer cells. MTT and colony formation assays showed that MCF-7-derived exosomes significantly increased the proliferation and clone-formation ability of breast cancer cells compared with MCF-10 A-derived exosomes (Fig. 2A and B). Furthermore, flow cytometry analysis revealed a lower percentage of apoptotic cells in the group treated with MCF-7-derived exosomes as compared to the group treated with MCF-10 A-derived exosomes (Fig. 2C). The wound healing assay and transwell assay demonstrated significant promotion of wound healing and invasion of breast cancer cells by MCF-7-derived exosomes, compared to MCF-10 A-derived exosomes (Fig. 2D and E). Additionally, the Western blot results indicated a substantial increase in expression of the mesenchymal markers N-cadherin and vimentin, accompanied by a decrease in expression of the epithelial marker E-cadherin in cells treated with MCF-7-derived exosomes (Fig. 2F), suggesting that MCF-7-derived exosomes may promote EMT in breast cancer. Therefore, our experimental data strongly supported the assertion that breast cancer-derived exosomes can enhance the proliferation, invasion, migration, and EMT of breast cancer cells.

### Exosomal circRHOT1 promotes the proliferation, wound healing, invasion, and EMT and inhibits the cell apoptosis of breast cancer cells in vitro

**Fig. 3:**
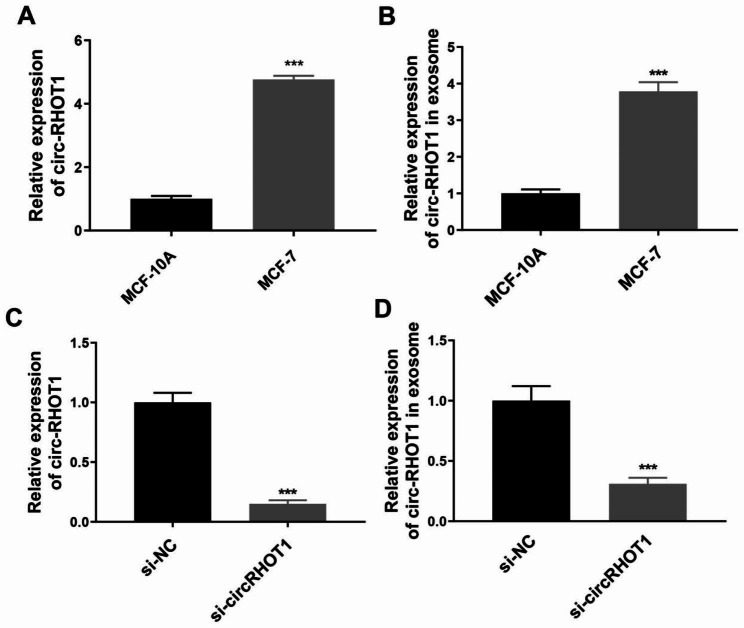
Expression of circRHOT1 in cells and exosomes. **A**, Expression of circRHOT1 in MCF-10 A and MCF-7 cells. **B**, Expression of exosomal circRHOT1 in MCF-10 A and MCF-7. **C**, Expression of circRHOT1 in MCF-7 cells after circRHOT1 knockdown. **D**, Expression of exosomal circRHOT1 in MCF-7 cells after circRHOT1 knockdown. * p < 0.05, ** p < 0.01, *** p < 0.001

To investigate the presence of circRHOT1 in exosomes derived from breast cancer cells, we initially assessed the expression levels of circRHOT1 in MCF-7 and MCF-10 A cell lines by using quantitative real-time PCR. As shown in Fig. 3A, circRHOT1 expression was notably higher in MCF-7 cells compared to MCF-10 A cells. In addition, circRHOT1 exhibited a significant upregulation in exosomes derived from MCF-7 cell culture supernatant (Fig. 3B). Subsequently, we utilized si-circRHOT1 to knock down the expression of circRHOT1 in MCF-7 cells. The quantitative real-time PCR analysis revealed a considerable decrease in circRHOT1 expression in both the cells (Fig. 3C) and exosomes (Fig. 3D) after circRHOT1 knockdown. Therefore, these results suggest the likelihood of circRHOT1 presence in the exosomes of breast cancer cells.

**Fig. 4:**
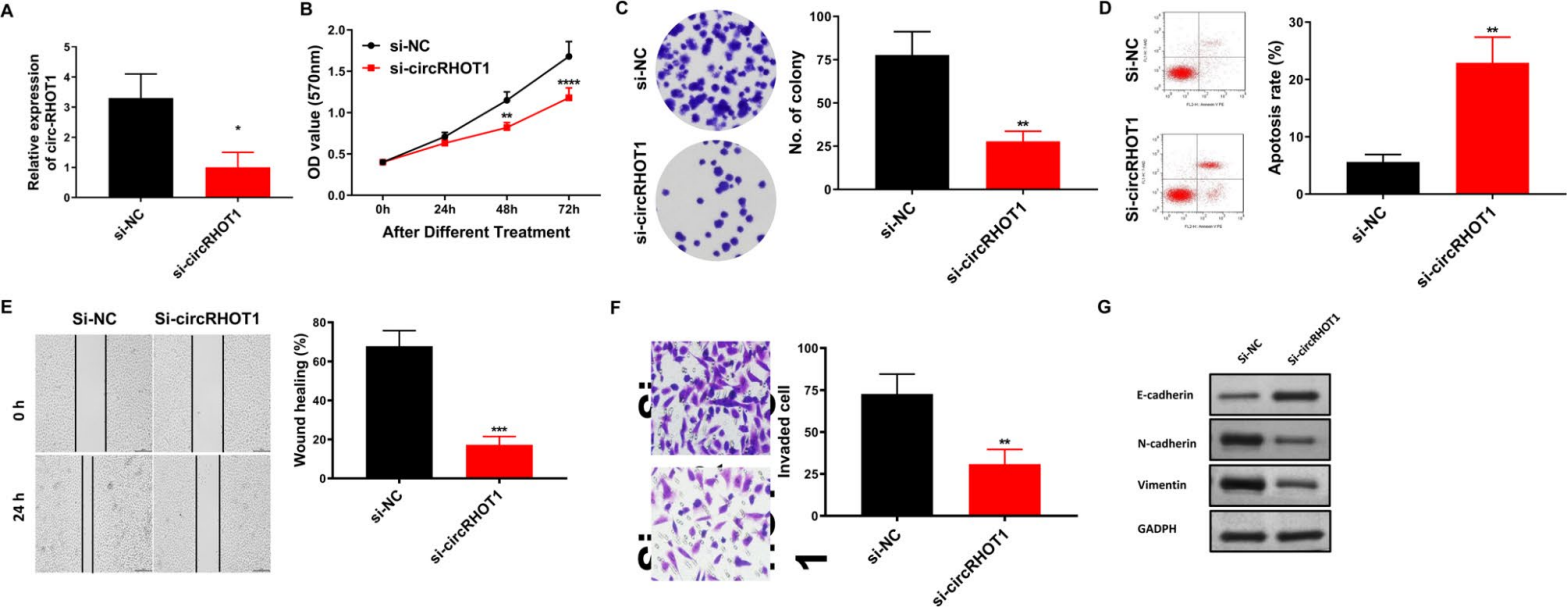
Exosomal circRHOT1 promoted proliferation, wound healing, invasion, and EMT but inhibited the cell apoptosis of breast cancer cells *in vitro* **A**, Expression of circRHOT1 in MCF-7 cells after co-culture with exosomes. **B**, Cell proliferation assessed by MTT assay. **C**, Colony formation assay results. **D**, Flow cytometry analysis of apoptosis. **E**, Wound healing assay results. **F**, Transwell assay of migration results. **G**, Western blot analysis of E-cadherin, N-cadherin, and vimentin expression. Compared with the si-NC group, ** p < 0.01, *** p < 0.001

To investigate that circRHOT1 is required for breast cancer progression, we extracted exosomes from culture supernatants of MCF-7 cells transfected with si-circRHOT1 and co-cultured them with untreated MCF-7 cells. The exosomes decreased the cicrRHOT1 expression in MCF-7 cells (Fig. 4A). MTT, colony formation assay, wound healing assay, and transwell invasion assay showed that the proliferation (Fig. 4B), colony formation ability (Fig. 4C), wound healing (Fig. 4E), and invasive ability (Fig. 4F) of MCF-7 cells treated with exosomes from si-circRHOT1-transfected cells were significantly reduced. Furthermore, flow cytometry results revealed that co-culture with exosomes from si-circRHOT1-transfected cells markedly increased the overall apoptosis rate of breast cancer cells (Fig. 4D). Given that EMT is the main mechanism of cancer metastasis, we investigated whether exosomal circRHOT1 affects the expression of EMT-related markers. Western blot showed that the expression of epithelial marker E-cadherin increased, while the expression of N-cadherin, and vimentin decreased after co-culture with exosomes from si-circRHOT1 cells for 24 h (Fig. 4G). These findings indicate that exosomal circRHOT1 can regulate the EMT and, consequently, regulate breast cancer progression. Collectively, these data suggest that circRHOT1 is present in breast cancer cell exosomes and that exosomal circRHOT1 can promote breast cancer cell proliferation, colony formation, invasion, metastasis, and EMT.

### CircRHOT1 functions as a mir-204-5p sponge

**Fig. 5:**
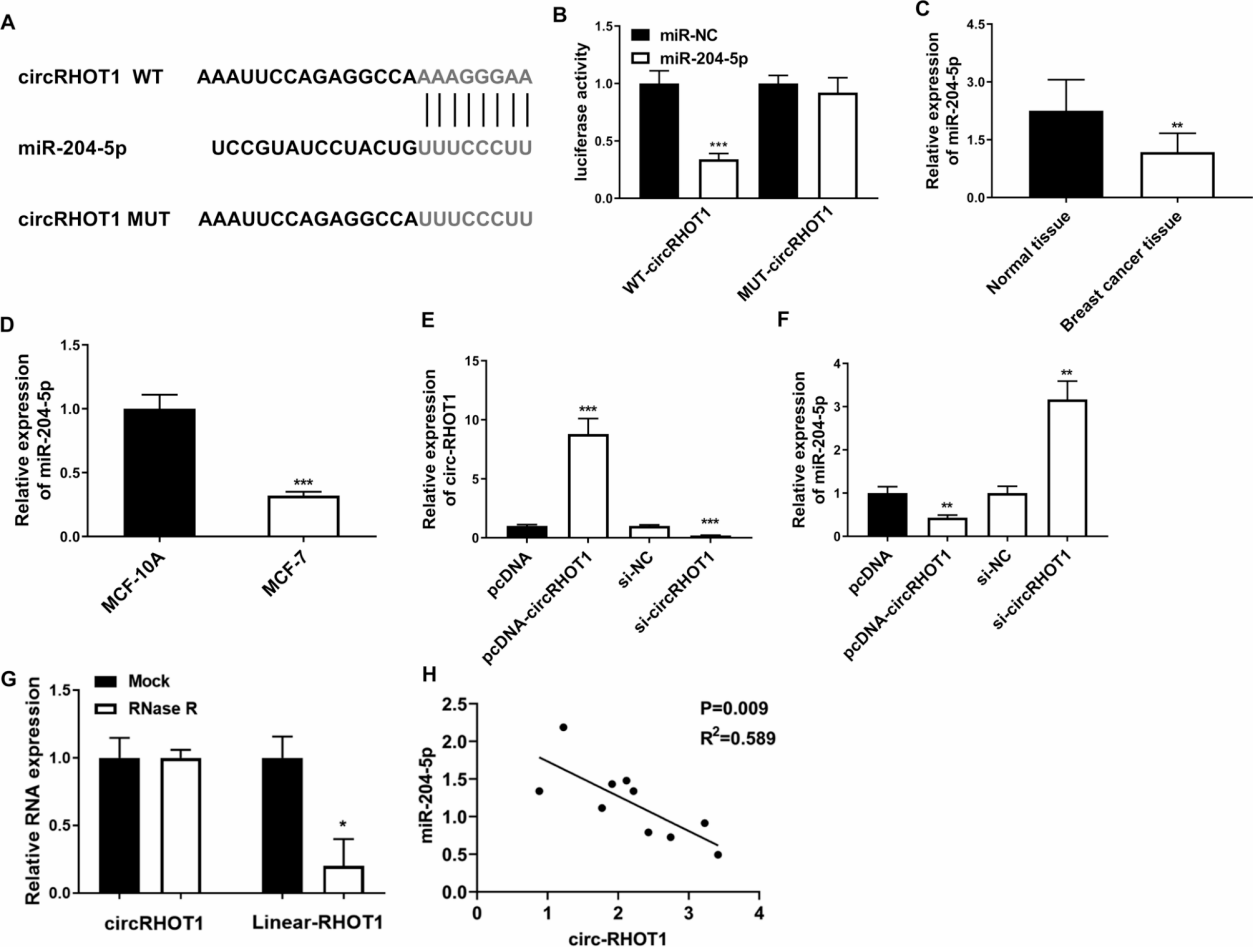
CircRHOT1 functions as a sponge of miR-204-5p. **A**, The potential interaction between circRHOT1 and miR-204-5p. **B**, The luciferase activities of WT-circRHOT1 and MUT circRHOT1 with miR-204-5p. **C**, Expression of miR-204-5p in normal control (n = 10) and breast cancer patients (n = 10). **D**, Expression of miR-204-5p in MCF-10 A and MCF-7. **E**, Expression of circRHOT1 after overexpression or knockdown. **F**, Expression of miR-204-5p after circRHOT1 overexpression or knockdown. **G**, Quantitative real-time PCR analysis of circRHOT1 after treatment with RNase R. **H**, Correlation between circ-RHOT1 and miR-204-5p. ** p < 0.01, *** p < 0.001

There is growing evidence suggesting that circRNAs act as miRNA sponges in tumor development and metastasis [11, 22, 28]. To explore the mechanism of circRHOT1 in breast cancer cells, we predicted the potential targeted miRNAs of circRHOT1 using the Circular RNA Interactome database. As shown in Fig. 5A, it was determined that miR-204-5p was a potential target for circRHOT1. To further confirm the target relationship between miR-204-5p and circRHOT1, a dual-luciferase reporter gene assay was performed. The results showed that compared with the miR-NC mimic, the miR-204-5p mimic group resulted in a significant downregulation of luciferase activity in cells transfected with wild-type circRHOT1, while the luciferase activity in cells transfected with mutant circRHOT1 was not significantly changed (Fig. 5B). Quantitative real-time PCR showed that the expression of miR-204-5p was significantly reduced in breast cancer patients and MCF-7 cells (Fig. 5 C and 5D). Furthermore, overexpression of circRHOT1 significantly reduced the expression of miR-204-5p, while knockdown of circRHOT1 significantly increased the expression of miR-204-5p (Fig. 5E F). We also confirmed the circular structure of circRHOT1 after digestion with RNase R (Fig. 5G). Additionally, Pearson’s correlation analysis was used to assess the correlation between circ-RHOT1 and miR-204-5p. The result showed that circ-RHOT1 exhibited a negative association with miR-204-5p (Fig. 5H). These results indicate that circRHOT1 could act as a sponge for miR-204-5p and reduce the expression of miR-204-5p.

### Exosomal circRHOT1 promotes the proliferation, migration, invasion, and EMT, and inhibits cell apoptosis of breast cancer cells via miR-204-5p

**Fig. 6:**
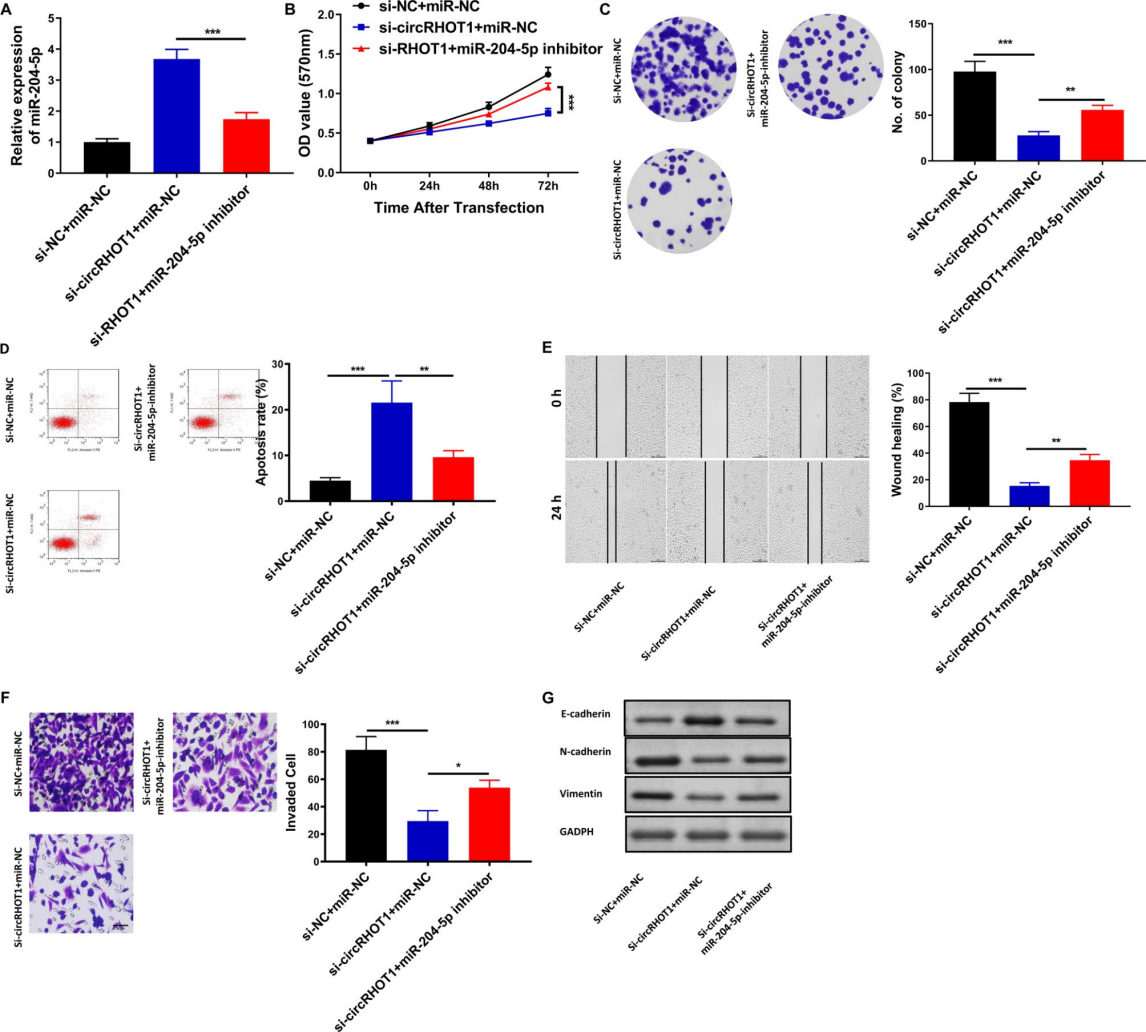
Exosomal circRHOT1 promoted the proliferation, migration, invasion, and, EMT but inhibited cell apoptosis of breast cancer cells via miR-204-5p. **A**, The expression of miR-204-5p among different treatment groups. **B**, Cell proliferation assessed by MTT assay. **C**, Colony formation assay results. **D**, Flow cytometry analysis of apoptosis. **E**, Wound healing assay results. **F**, Transwell assay of migration results. **G**, Western blot analysis of E-cadherin, N-cadherin, and vimentin expression. Compared with si-NC + miR-NC and si-circRHOT1 + miR-NC group, *P < 0.05, ** p < 0.01, *** p < 0.001

First, we extracted exosomes from culture supernatants of MCF-7 cells transfected with si-circRHOT1 or si-NC. Then, MCF-7 cells were intervented with these exosomes and miR-204-5p inhibitors. The results showed that miR-204-5p inhibitor rescued the effects of these exosomes on cell growth, wound healing, apoptosis, and cell invasion (Fig. 6A F). In addition, miR-204-5p inhibitor also rescued the expression of E-cadherin, N-cadherin, and vimentin induced by these exosomes (Fig. 6G).

### Exosomal circRHOT1 promotes Breast cancer progression through the miR-204-5p/PRMT5 axis

**Fig. 7:**
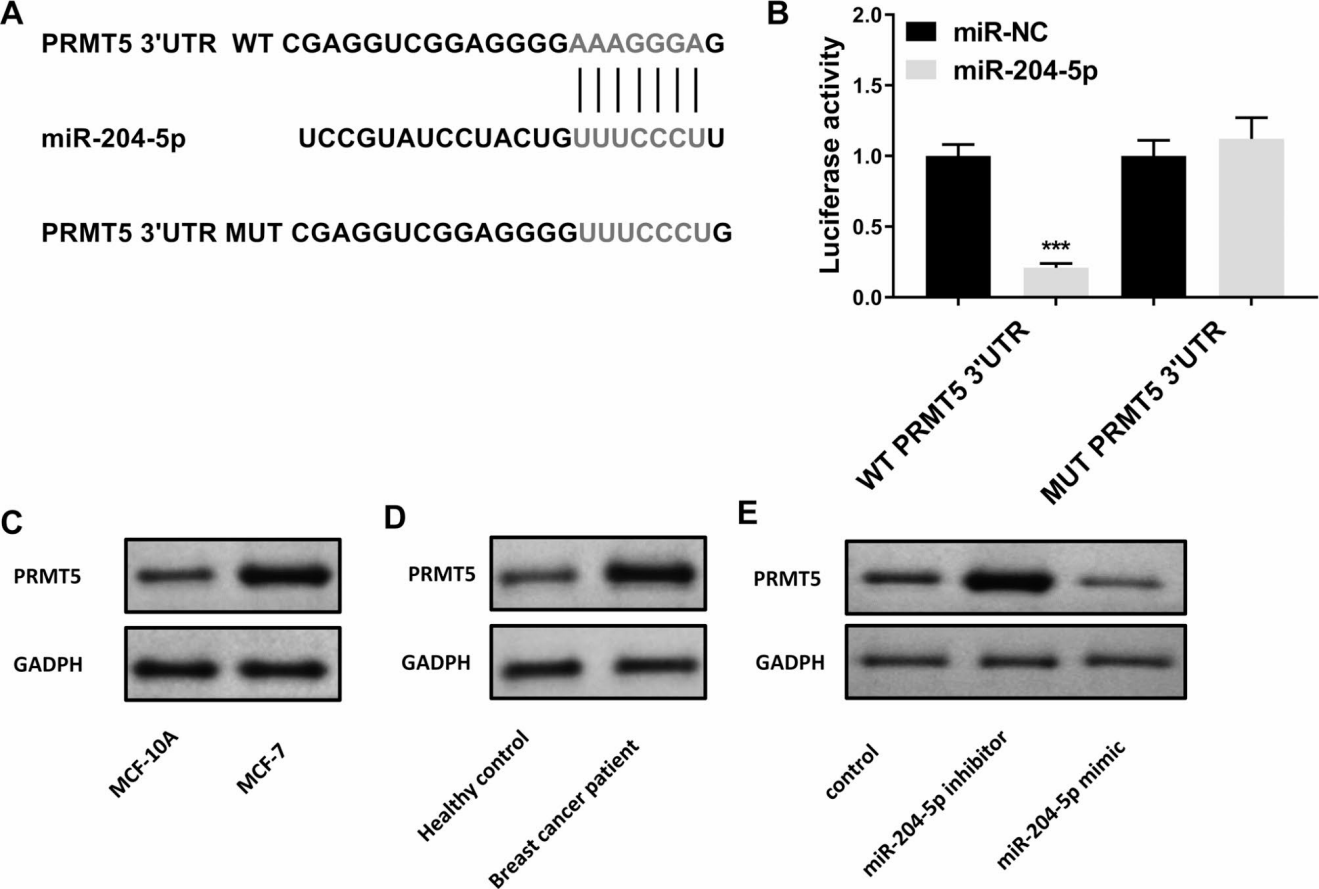
MiR-204-5p regulated the expression of PRMT5. **A**, The potential interaction between miR-204-5p and PRMT5. **B**, The luciferase activities of WT-PRMT5 and MUT PRMT5 with miR-204-5p. **C**, Expression of PRMT5 in MCF-10 A and MCF-7. **D**, Expression of PRMT5 in healthy control (n = 3) and breast cancer patients (n = 3). **E**, Expression of PRMT5 after treatment with miR-204-5p inhibitor or mimic. Compared with control and miR-204-5p mimic group, * p < 0.05, ** p < 0.01, *** p < 0.001

It has been demonstrated that miRNAs can target downstream genes by binding to the 3’ UTR [40]. In this study, we utilized TargetScan to predict that the PRMT5 3’ UTR may be the target by miR-204-5p (Fig. 7A). Similarly, through the use of a dual-luciferase reporter gene assay, we provided evidence that treatment with miR-204-5p mimic substantially decreased the luciferase activity in cells transfected with wild-type PRMT5, but not in cells transfected with mutant PRMT5 (Fig. 7B). Moreover, Western blot showed that PRMT5 expression levels were increased in breast cancer patients and MCF-7 cells (Fig. 7 C and 7D). Additionally, miR-204-5p inhibitors upregulated PRMT5 expression, while miR-204-5p mimics downregulated PRMT5 expression (Fig. 7E). These results indicate that miR-204-5p negatively regulates PRMT5 expression.

**Fig. 8:**
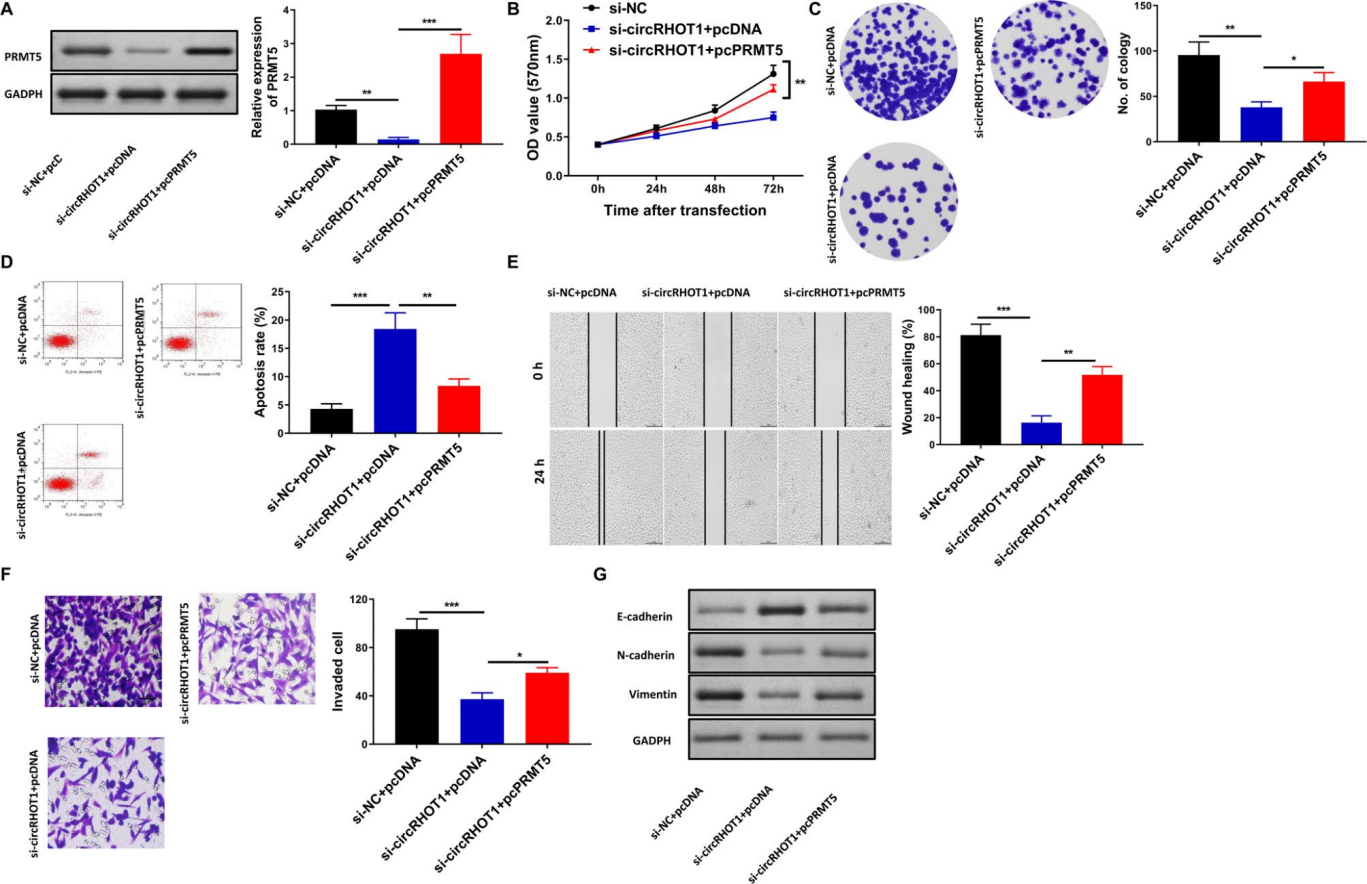
Exosomal circRHOT1 promoted the proliferation, migration, and invasion of MCF-7 via PRMT5. **A**, The expression of PRMT5 among different treatment groups. **B**, Cell proliferation assessed by MTT assay. **C**, Colony formation assay results. **D**, Flow cytometry analysis of apoptosis. **E**, Wound healing assay results. **F**, Transwell assay of migration results. **G**, Western blot analysis of E-cadherin, N-cadherin, and vimentin expression. Compared with si-NC + pcDNA and si-circRHOT1 + pcDNA group, *P < 0.05, ** p < 0.01, *** p < 0.001

Additionally, to further confirm whether circRHOT1 promotes breast cancer progression through the miR-204-5p/PRMT5 axis, we overexpressed PRMT5 in MCF-7 cells and then treated the cells with exosomes derived from MCF-7 cells transfected with si-circRHOT1 or si-NC. MTT, colony formation assay, flow cytometry analysis of apoptosis, wound healing assay, and Transwell invasion assay showed that PRMT5 overexpression could reverse the effect of exosomes from MCF-7 cells transfected with si-circRHOT1 on cell growth, apoptosis, wound healing, and cell invasion (Fig. 8A F). In addition, PRMT overexpression could rescue the alterations in E-cadherin, N-cadherin, and vimentin induced by exosomes from MCF-7 cells transfected with si-circRHOT1, thus reversing EMT (Fig. 8G).

### Exosomal circRHOT1 promotes the tumor growth of breast cancer in vivo

**Fig. 9:**
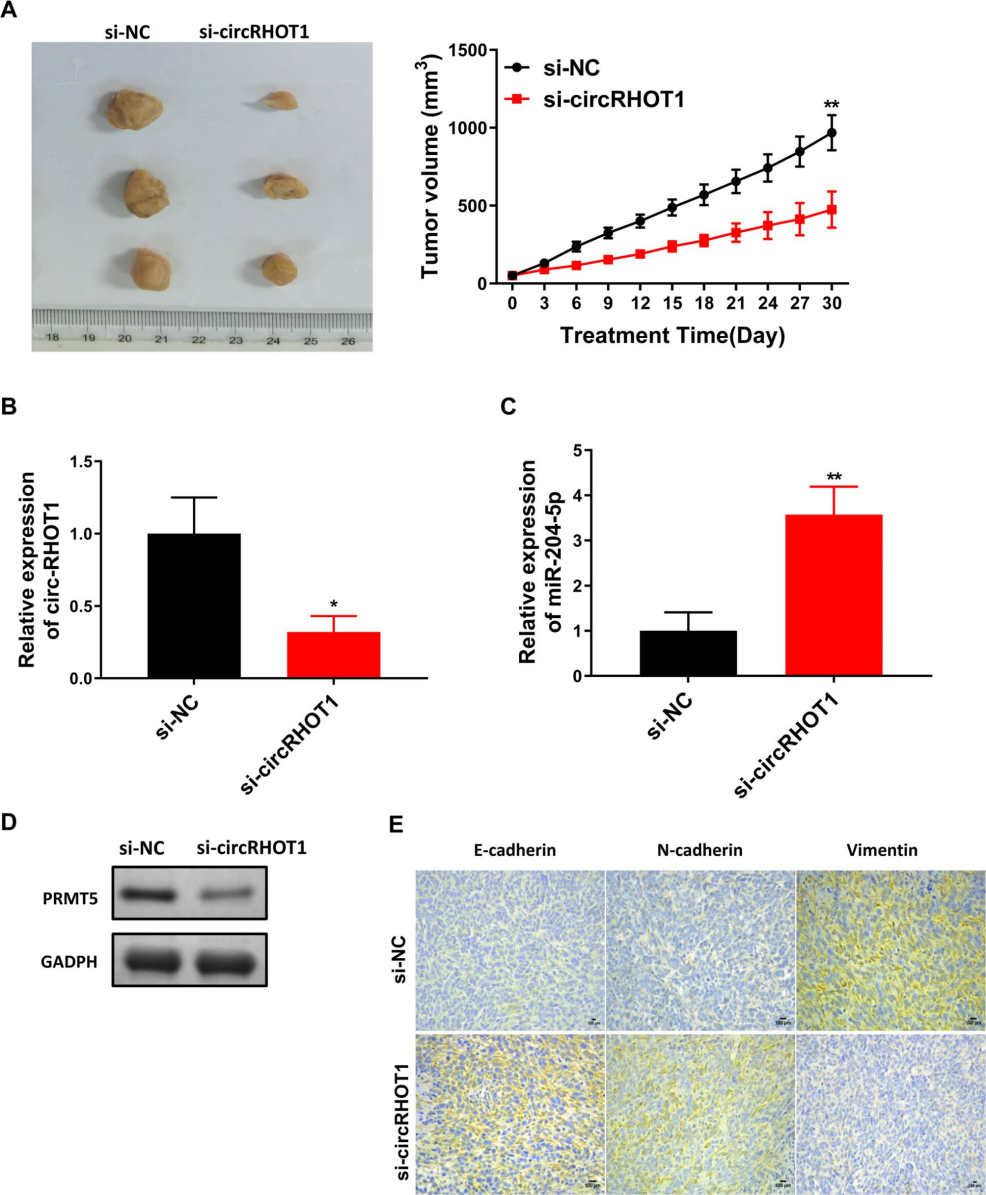
Exosomal circRHOT1 promoted the tumor growth of breast cancer in vivo. **A**, Representative images of the tumor at day 30 and tumor volume over time. **B**, The expression of circRHOT1 in tumor tissue of si-NC and si-circRHOT1 group. **C**, The expression of miR-204-5p in tumor tissue of si-NC and si-circRHOT1 group. **D**, The expression of circRHOT1 in the tumor tissue of si-NC and si-circRHOT1 group. **E**, The expression of E-cadherin, N-cadherin, and vimentin expression by immunohistochemistry. Compared with si-NC group, *P < 0.05, ** p < 0.01, *** p < 0.001

We finally investigated the effect of exosomal circRHOT1 on breast cancer development in vivo by constructing a tumor-bearing mouse model. The results showed that mice in the si-circRHOT1 group had significantly smaller tumor volumes compared to those in the control group (Fig. 9A). This finding indicated that the tumor growth of breast cancer cells in vivo was inhibited after exosomes (from cells transfected with si-circRHOT1 ) injection. In addition, Western blot and quantitative real-time PCR analysis showed that the expression levels of PRMT5 and circRHOT1 were decreased in tumor tissues of the si-circRHOT1 group compared with the si-NC group, while the expression level of miR-204-5p was increased (Fig. 9B and D). Finally, immunohistochemistry analysis showed that exosomes from cells transfected with si-circRHOT1 prompted an increase in the expression of E-Cadherin and N-cadherin and induced a decrease in Vimentin (Fig. 9E), thus inhibiting the EMT of breast cancer cells. Taken together, the above results further confirm that exosomal circRHOT1 may promote breast cancer growth in vivo via miR-204-5p/PRMT5 axis.

## Discussion

Exosomes are nano-sized membrane vesicles released by various cells and are thought to play crucial roles in intercellular communications. In breast cancer, exosomes are emerging as significant mediators of oncogenic information at both local and systemic levels through the horizontal transfer of diverse bioactive molecules, including proteins and mRNAs. This active involvement of exosomes contributes to cancer progression [41]. Wu et al. found that exosomal miRNAs facilitated intercellular communication and promoted the cell migration and metastasis of breast cancer [42]. Xing et al. found that exosomal miRNA could promote breast cancer metastases to the brain [43]. This study involved the isolation and identification of breast cancer-derived exosomes using electron microscopy and Western blot. Subsequently, these exosomes were co-cultured with MCF-7 cells. The findings demonstrated that the breast cancer-derived exosomes could promote the proliferation, migration, and invasion of MCF-7. These results reinforce the significant contribution of exosomes in the development of cancer. Besides, we examined the expression of exosomal circRHOT1 and found that circRHOT1 was markedly upregulated in the serum of breast patients as well as in breast cancer cells. Considering its elevated expression in breast cancer, exosomal circRHOT1 may hold the potential as a diagnostic biomarker for breast cancer. More importantly, we isolated exosomes from culture supernatants of MCF-7 cells transfected with si-circRHOT1 and co-cultured them with untreated MCF-7 cells. Our data indicate that suppression of circRHOT1 could inhibit the proliferation, migration, and invasion of MCF-7 cells. These results were consistent with previous reports on the role of circRNA in cancers [27, 35]. Collectively, the data suggest that circRHOT1 is present in breast cancer cell exosomes and that exosomal circRHOT1 can promote breast cancer cell proliferation, colony formation, invasion, metastasis, and EMT.

CircRNAs, a class of endogenous non-coding RNAs, exhibit abundant and stable expression [12]. They play important roles in the progression and development of various cancers [44, 45], functioning as miRNA sponges. For instance, circRHOT1 has been reported to mediate the cell proliferation, apoptosis, and invasion of pancreatic cancer cells by sponging miR-125a-3p [28]. It can also participate in chondrocyte autophagy and proliferation in osteoarthritis by interacting with miR-142-5p [46]. Additionally, circRHOT1 could promote the progression of breast cancer by regulating miR-106a-5p/STAT3 [38]. In this study, we performed bioinformatics analysis and dual-luciferase reporter assays to investigate the mechanism of exosomal circRHOT1 in breast cancer cells. Our analysis revealed that exosomal circRHOT1 may interact with miR-204-5p, which was known to be downregulated in breast cancer patients and MCF-7 cells. Overexpression of circRHOT1 significantly reduced miR-204-5p expression; on the contrary, knockdown of circRHOT1 significantly increased miR-204-5p expression. Further mechanistic investigations revealed that exosomal circRHOT1 could act as a miR-204-5p sponge and exert pro-cancer effects. Moreover, miR-204-5p inhibitor reversed the effect of exosomal circRHOT1 knockdown on cell growth, wound healing, apoptosis, and cell invasion as well as on the expression of E-cadherin, N-cadherin, and vimentin. These findings provided evidence that exosomal circRHOT1 potentially played a crucial role in the cell proliferation of breast cancer cells in vitro and in vivo via miR-204-5p, suggesting its significance as a key regulator in breast cancer progression. However, the constraint of limited sample size poses a limitation factor in our study.

Previous studies have demonstrated that miR-204-5p functions as a tumor suppressor in the development of various human tumors, including breast cancer [47–49]. PRMT5 regulates breast cancer cell growth through epigenetic silencing of DKK1 and DKK3, which are WNT/β-CATENIN pathway antagonists, thus resulting in up-regulation of WNT/β-CATENIN proliferative signaling [50]. However, the association between PRMT5 and miR-204-5p has not been reported. Herein, we found that miR-204-5p could directly target PRMT5 using bioinformatics tools and dual-luciferase gene reporter assay. Interestingly, in contrast to the miR-204-5p expression pattern, PRMT5 was overexpressed in breast cancer. After treatment with miR-204-5p inhibitors, PRMT5 expression was upregulated, while after treatment with miR-204-5p mimics, PRMT5 expression was downregulated. These results indicated that miR-204-5p could negatively regulate PRMT5 expression. Furthermore, PRMT5 overexpression reversed the effect of circRHOT1 knockdown on cell growth, apoptosis, wound healing, and cell invasion, as well as on E-cadherin, N-cadherin, and vimentin expression, thus reversing EMT. Taken together, all data shows that exosomal circRHOT1 promoted breast cancer proliferation, invasion, metastasis, and EMT through regulation of the miR-204-5p/PRMT5 axis.

## Conclusions

In conclusion, our findings reveal that exosomal circRHOT1 plays crucial roles in the progression of breast cancer through the miR-204-5p/PRMT5 axis. These findings provide novel insights into the mechanism by which circRHOT1 promotes the development of breast cancer. Moreover, circRHOT1 and miR-204-5p may serve as potential targets for breast cancer treatment.

### Electronic supplementary material

Below is the link to the electronic supplementary material.

Supplementary Material 1

## Data Availability

The datasets generated and analyzed for the current study are available from the corresponding author upon reasonable request.

## Abbreviations

circRHOT1: Circular RNA RHOT1
PRMT5: protein arginine methyltransferase 5
CircRNAs: Circular RNAs
EMT: epithelial-mesenchymal transition
miRNA: microRNA
EVs: extracellular vesicles
