# Supplementary material for: Exosomal circRNA RHOT1 promotes breast cancer progression by targeting miR-204-5p/ PRMT5 axis
Source: Cancer Cell Int. 2023 Nov 3;23:260. doi: 10.1186/s12935-023-03111-5 (PMC10623849; doi:10.1186/s12935-023-03111-5)
Supplement: Supplementary file 1 — Supplementary Material 1 [file 12935_2023_3111_MOESM1_ESM.ppt]

## Slide 1
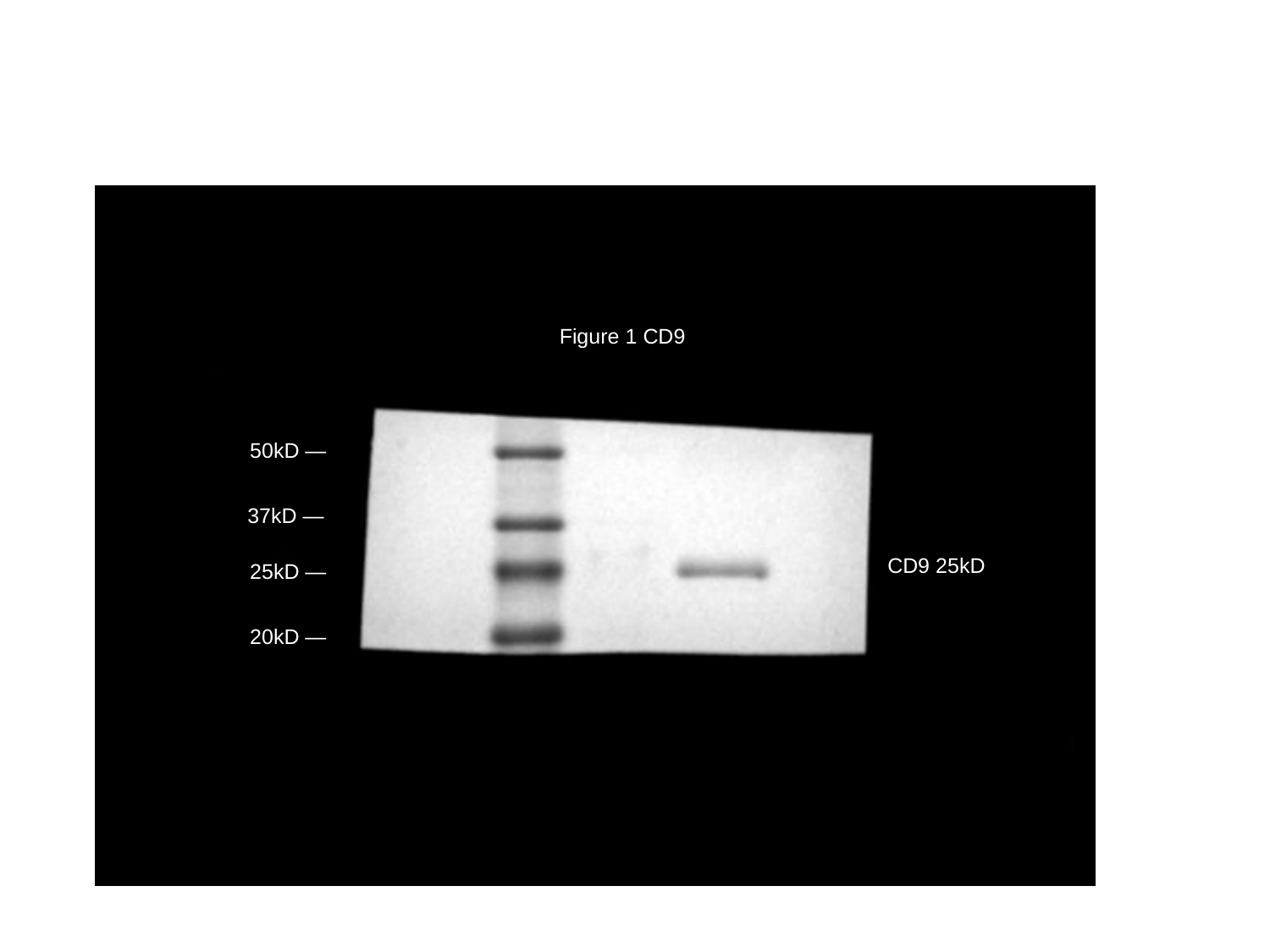

Figure 1 CD9
50kD —
37kD —
CD9 25kD
25kD —
20kD —

## Slide 2
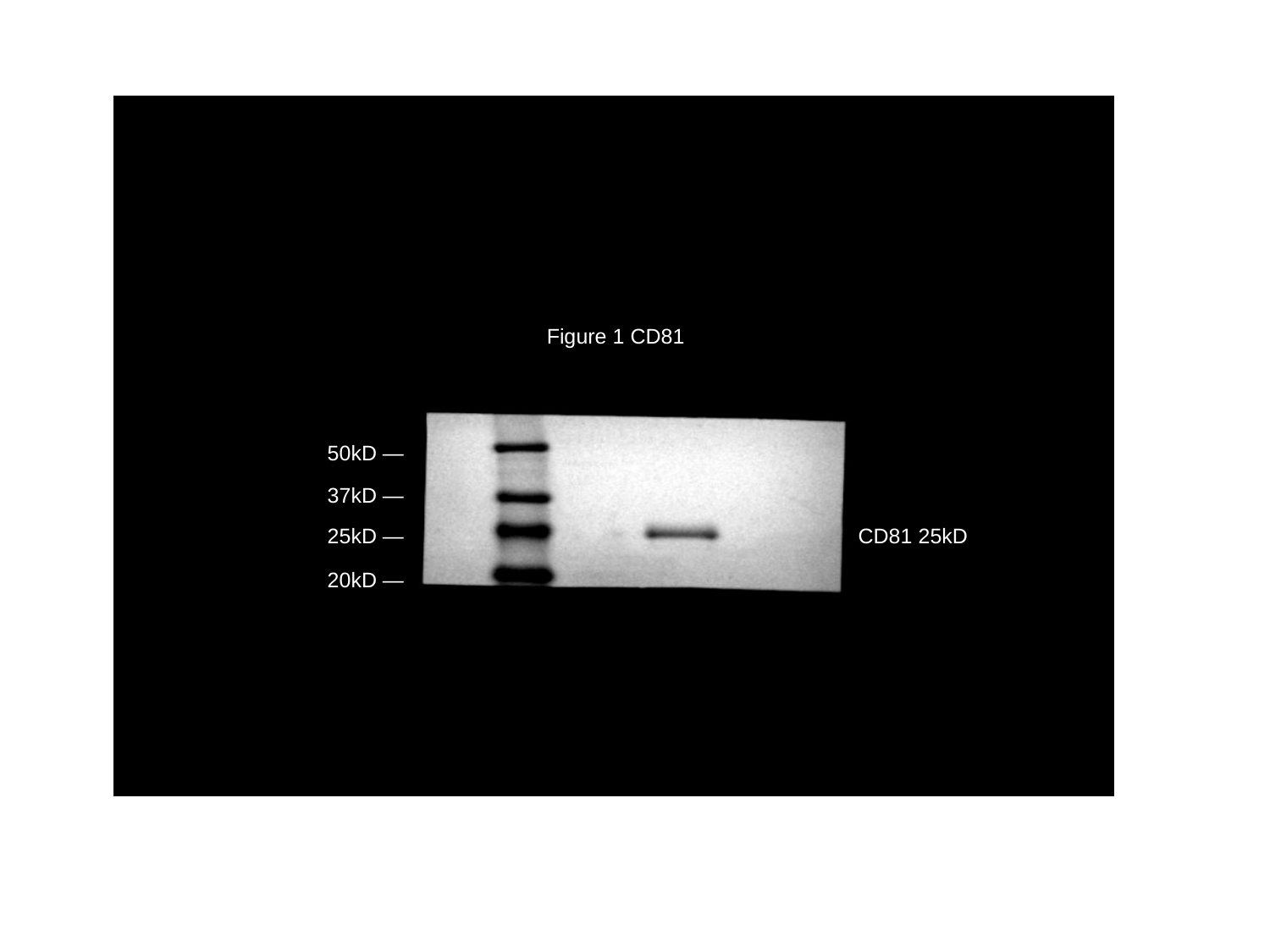

Figure 1 CD81
50kD —
37kD —
25kD —
CD81 25kD
20kD —

## Slide 3
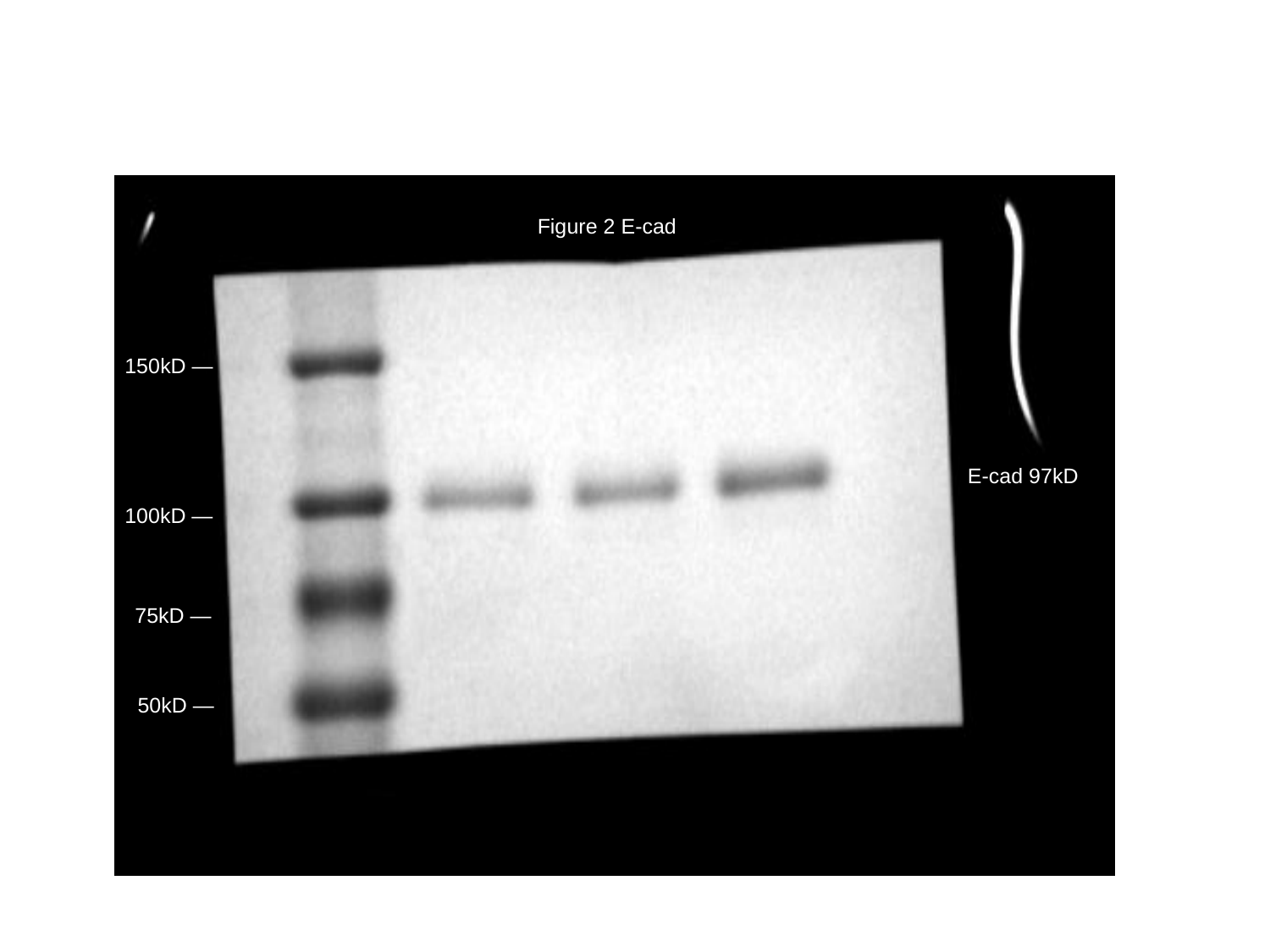

Figure 2 E-cad
150kD —
E-cad 97kD
100kD —
75kD —
50kD —

## Slide 4
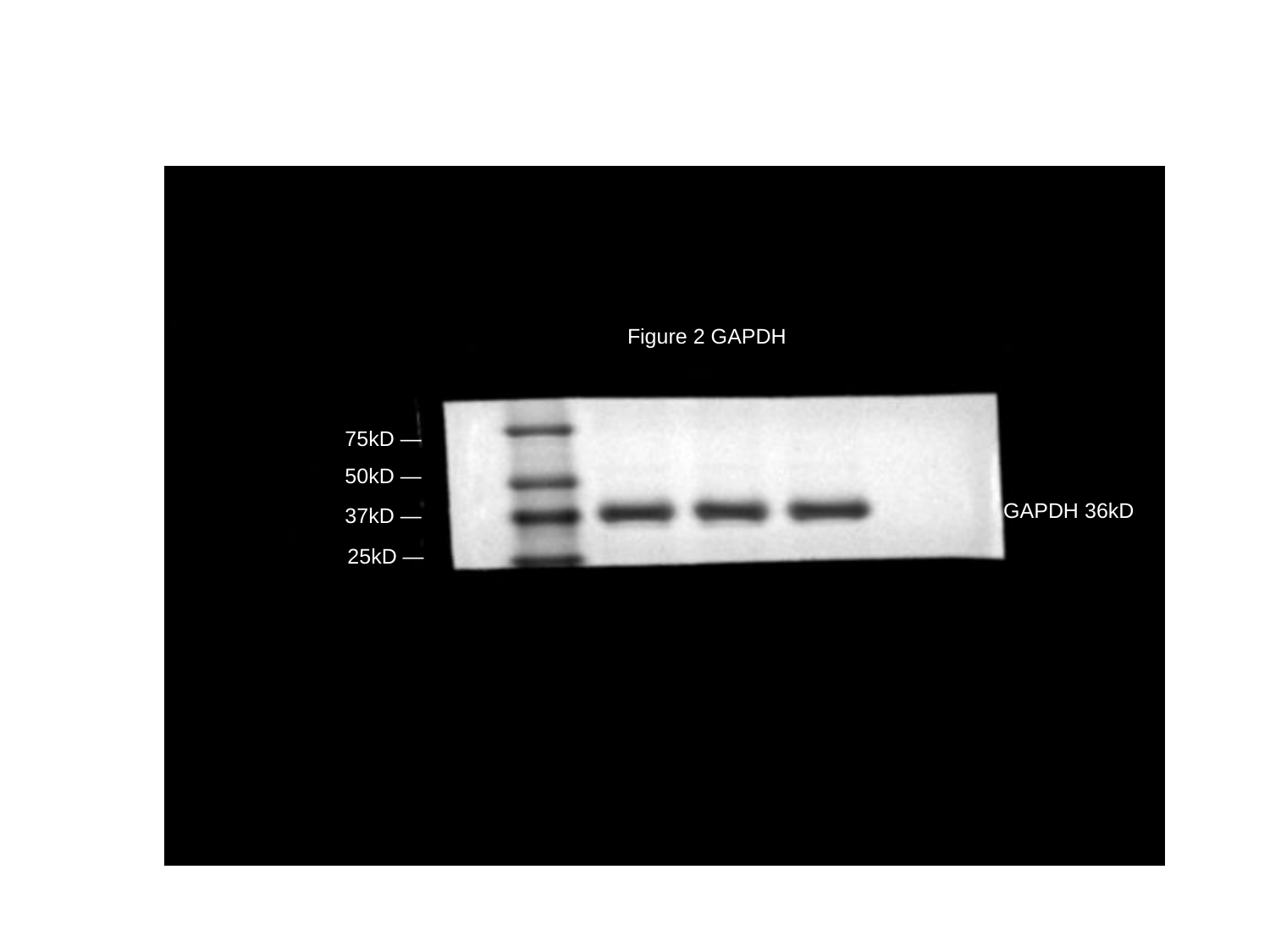

Figure 2 GAPDH
75kD —
50kD —
 GAPDH 36kD
37kD —
25kD —

## Slide 5
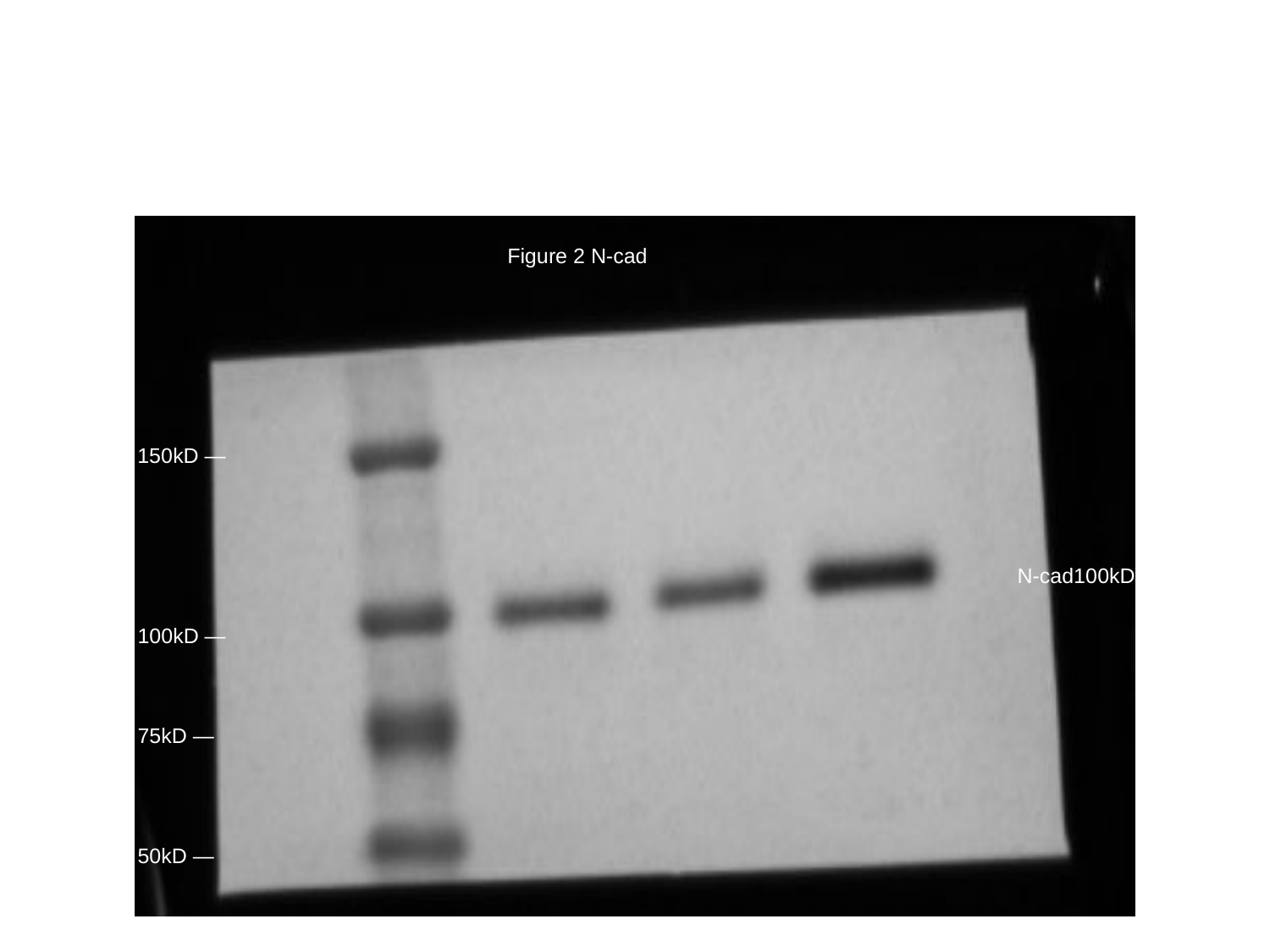

Figure 2 N-cad
150kD —
N-cad100kD
100kD —
75kD —
50kD —

## Slide 6
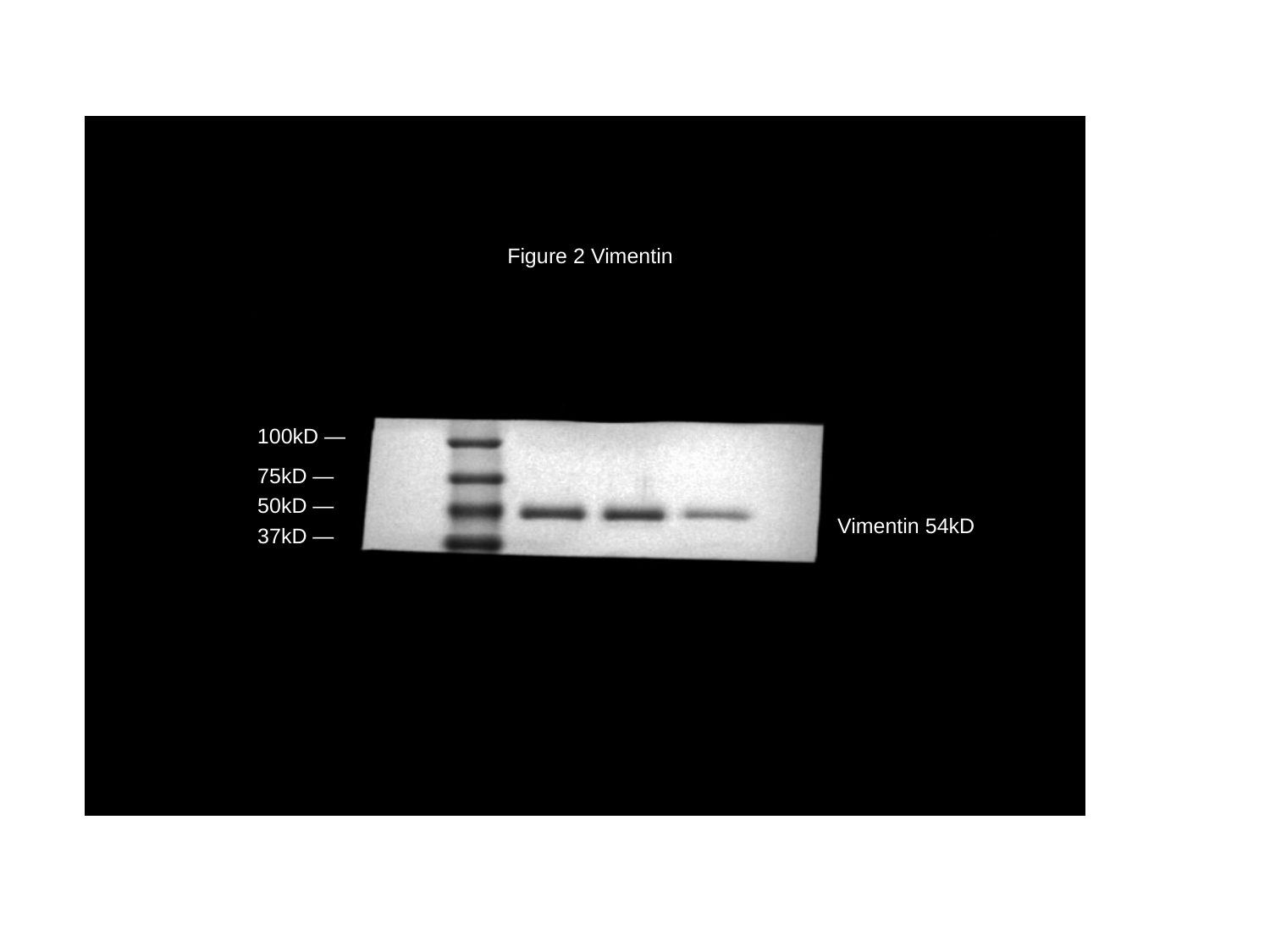

Figure 2 Vimentin
100kD —
75kD —
50kD —
Vimentin 54kD
37kD —

## Slide 7
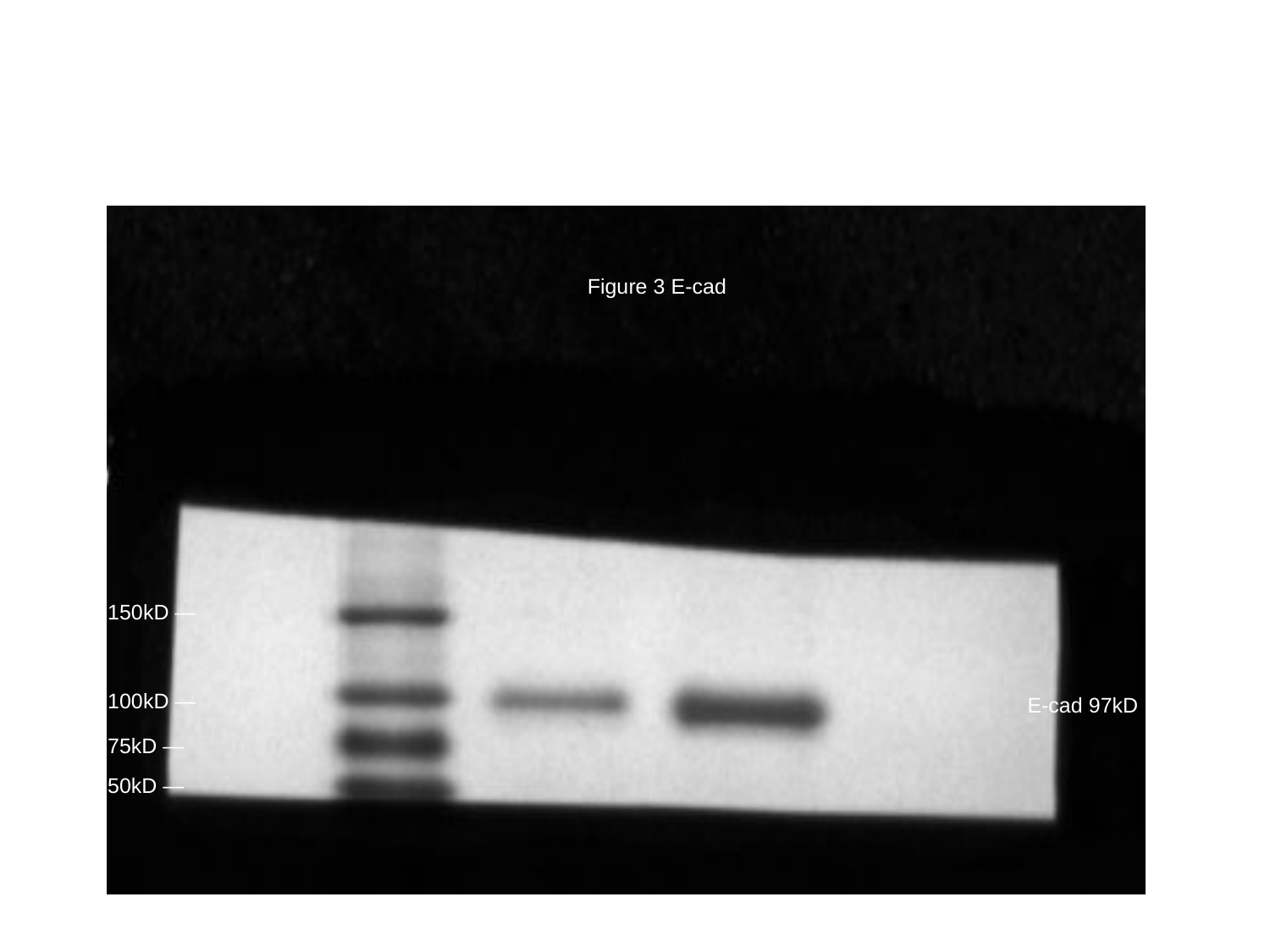

Figure 3 E-cad
150kD —
100kD —
E-cad 97kD
75kD —
50kD —

## Slide 8
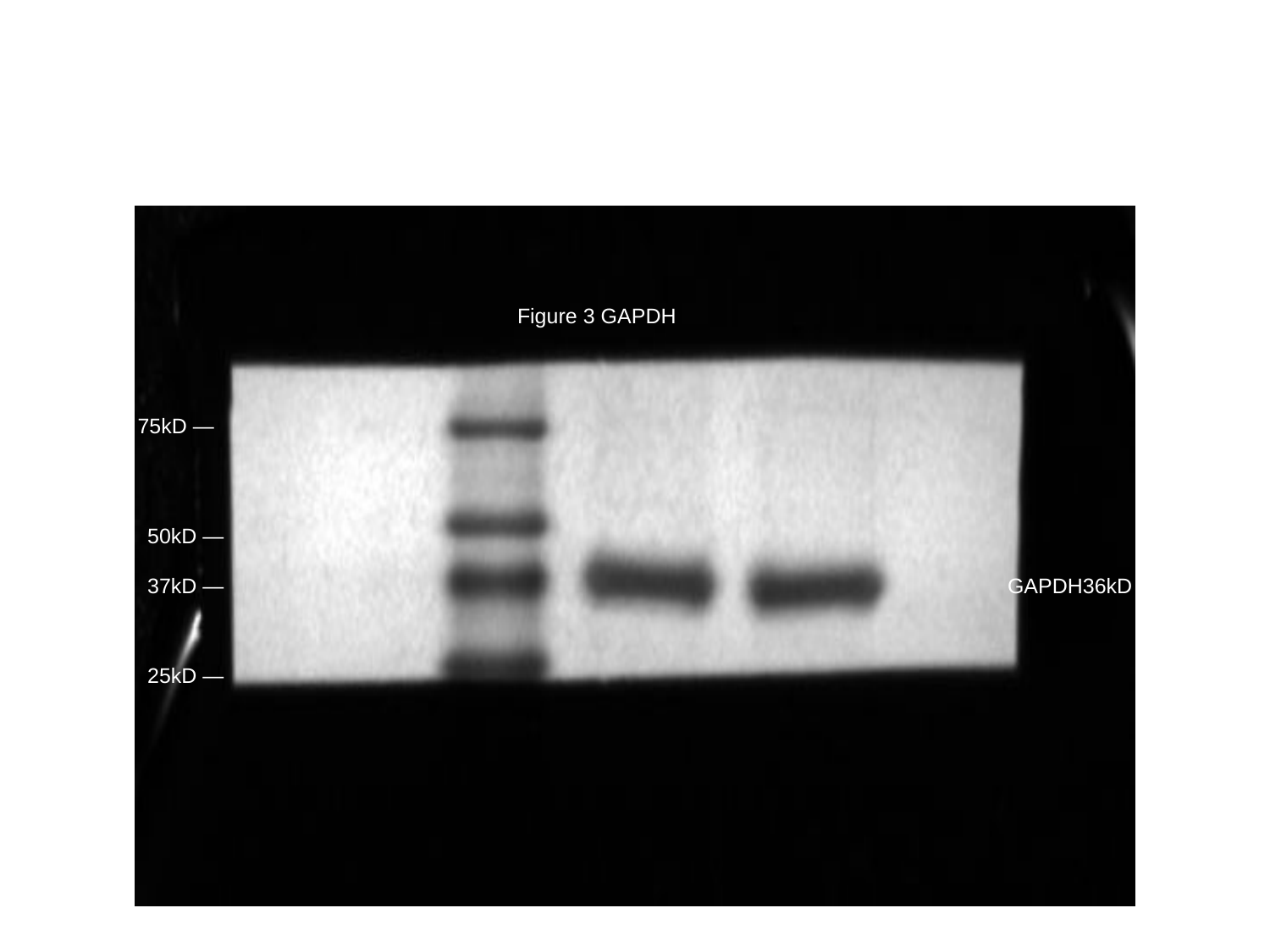

Figure 3 GAPDH
75kD —
50kD —
37kD —
GAPDH36kD
25kD —

## Slide 9
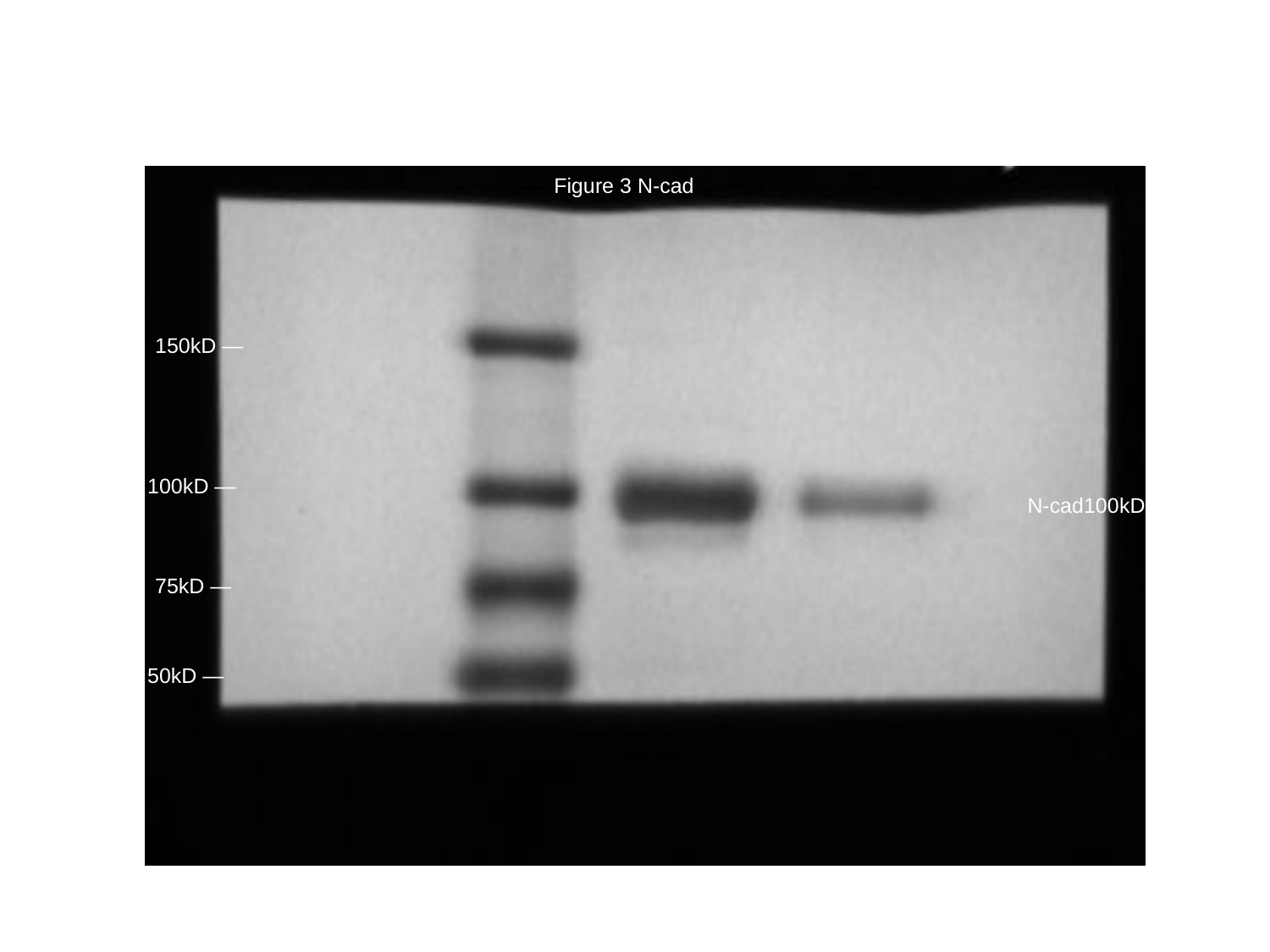

Figure 3 N-cad
150kD —
100kD —
N-cad100kD
75kD —
50kD —

## Slide 10
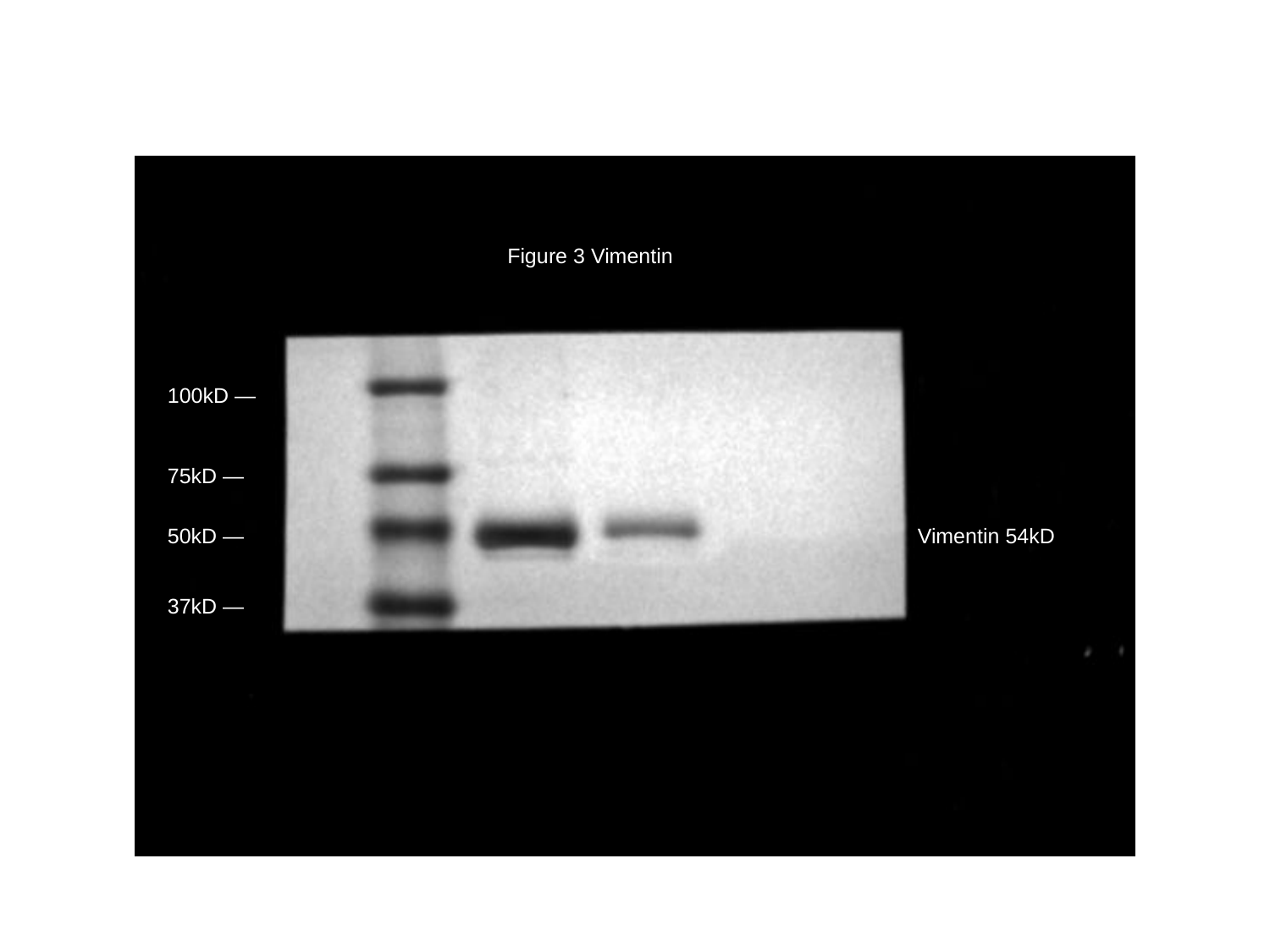

Figure 3 Vimentin
100kD —
75kD —
50kD —
Vimentin 54kD
37kD —

## Slide 11
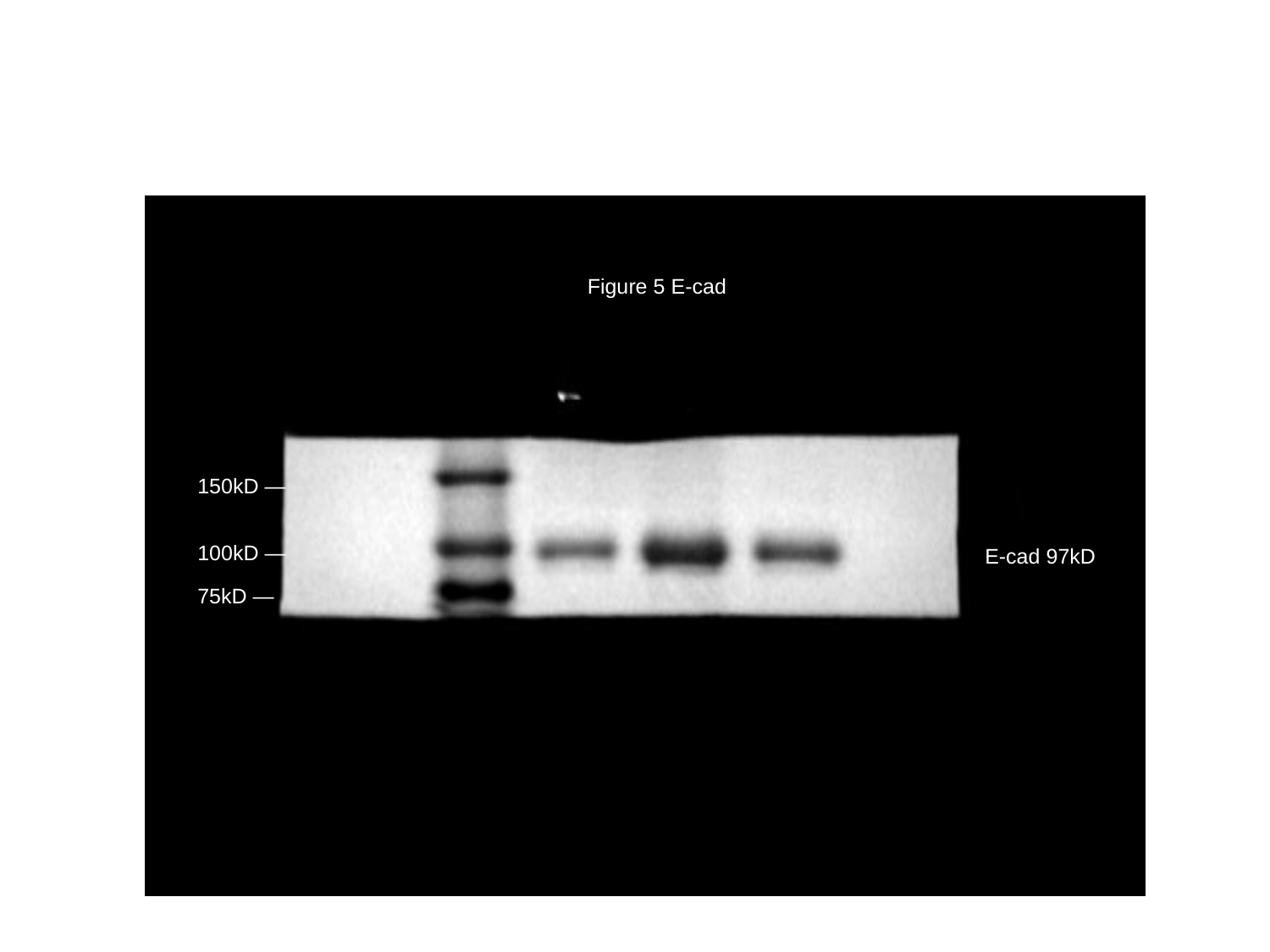

Figure 5 E-cad
150kD —
100kD —
E-cad 97kD
75kD —

## Slide 12
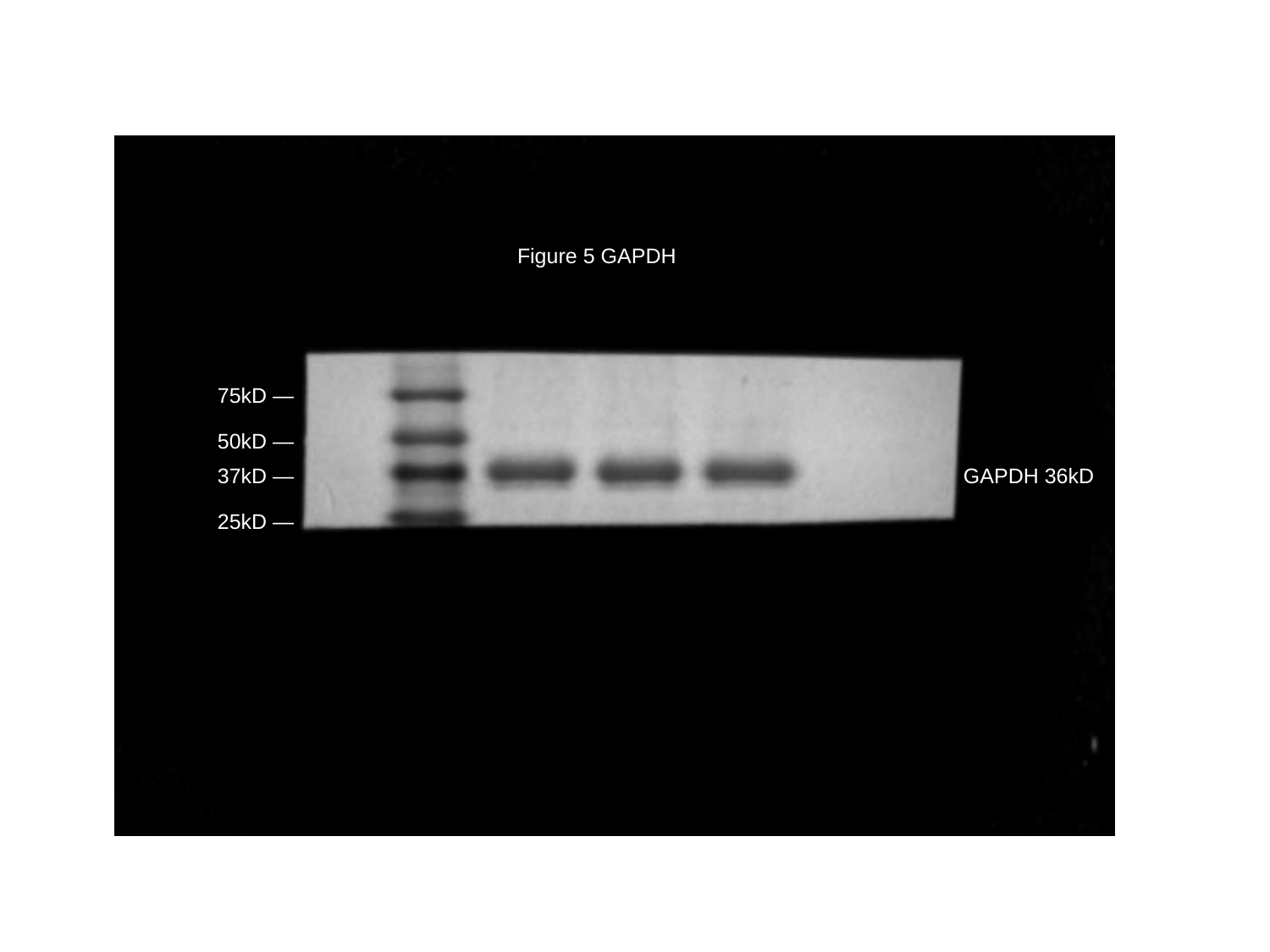

Figure 5 GAPDH
75kD —
50kD —
37kD —
 GAPDH 36kD
25kD —

## Slide 13
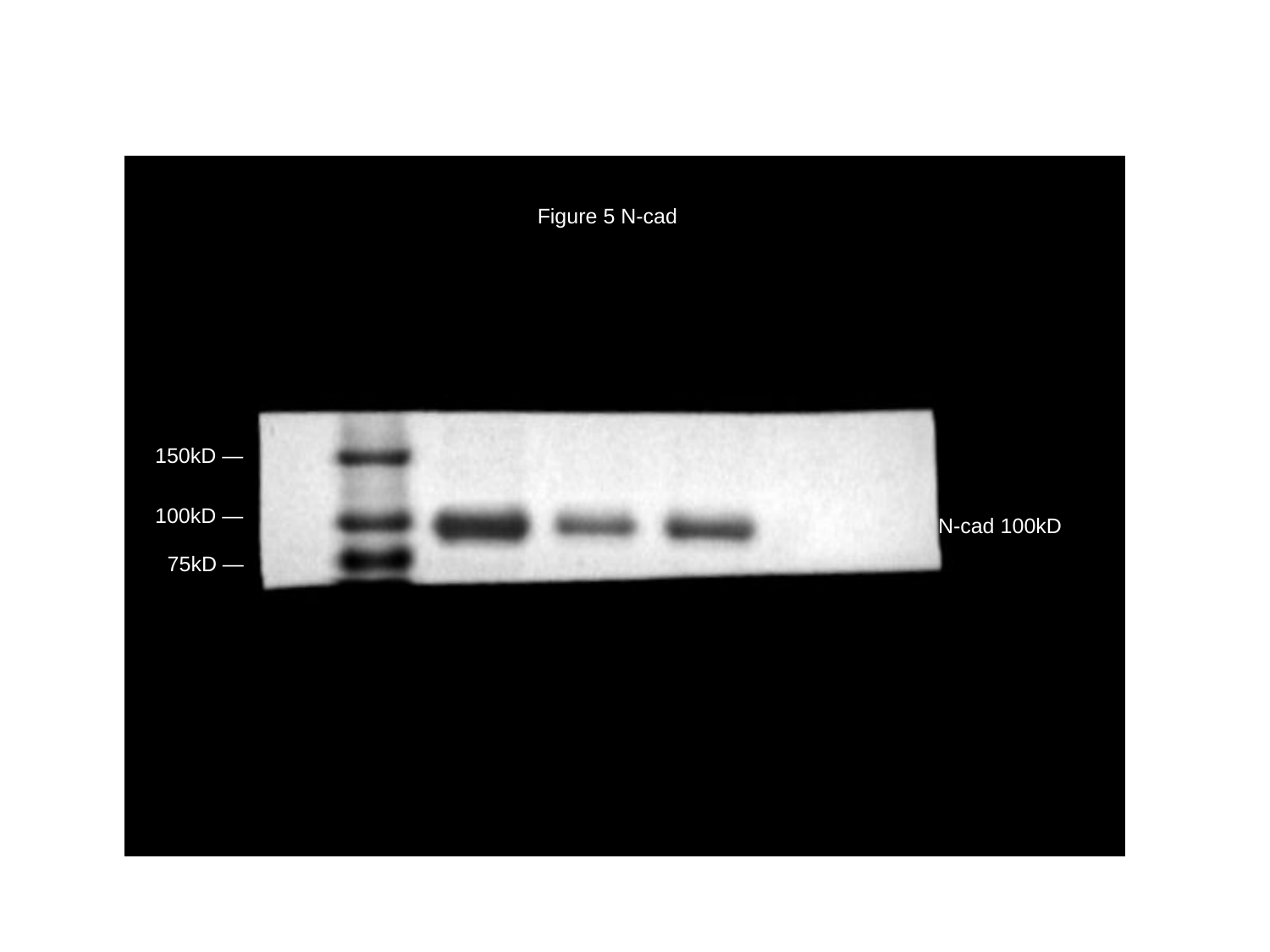

Figure 5 N-cad
150kD —
100kD —
N-cad 100kD
75kD —

## Slide 14
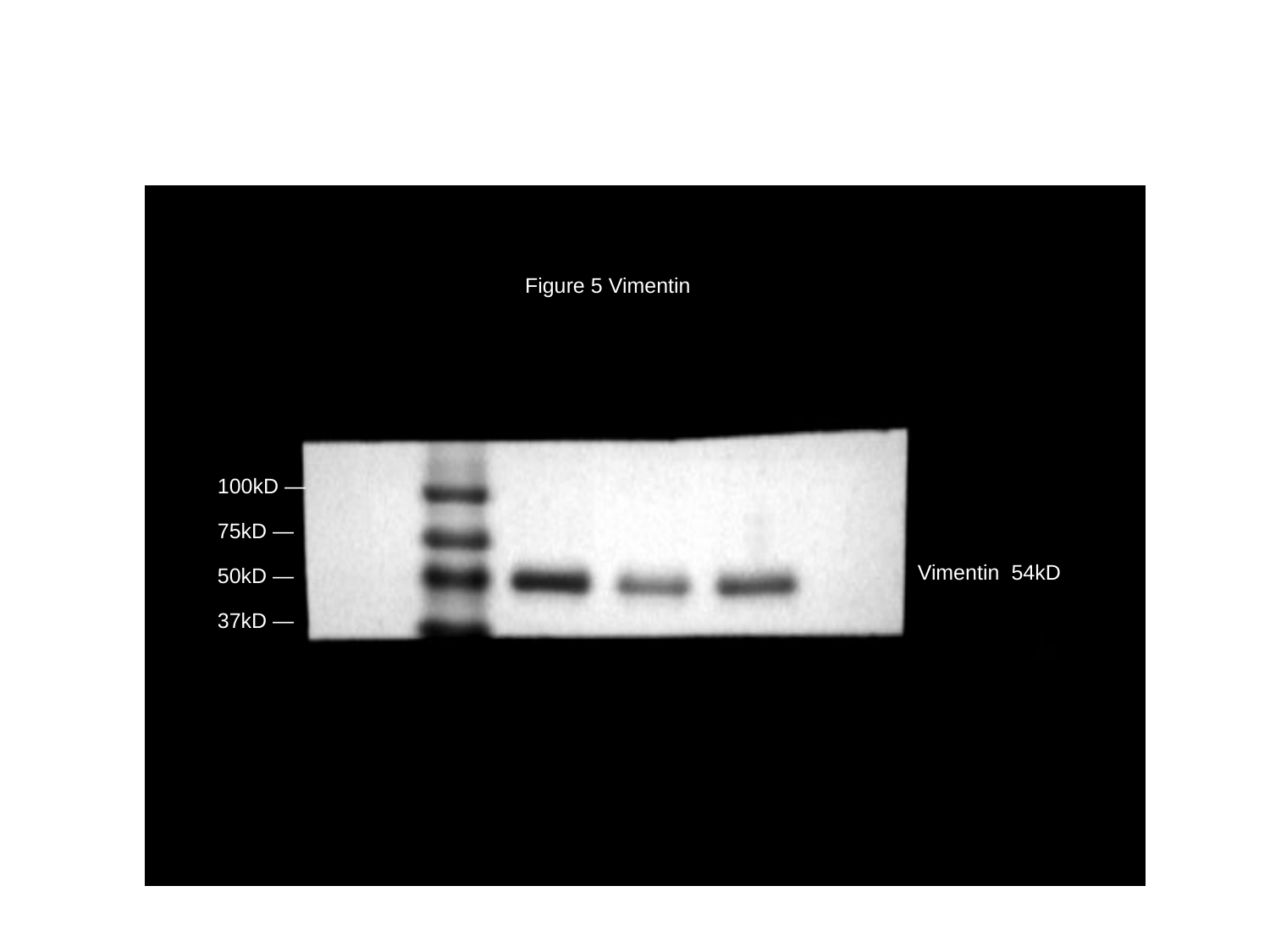

Figure 5 Vimentin
Figure 5 Vimentin
95kD —
72kD —
100kD —
Vimentin 54kD
75kD —
55kD —
Vimentin 54kD
50kD —
43kD —
37kD —

## Slide 15
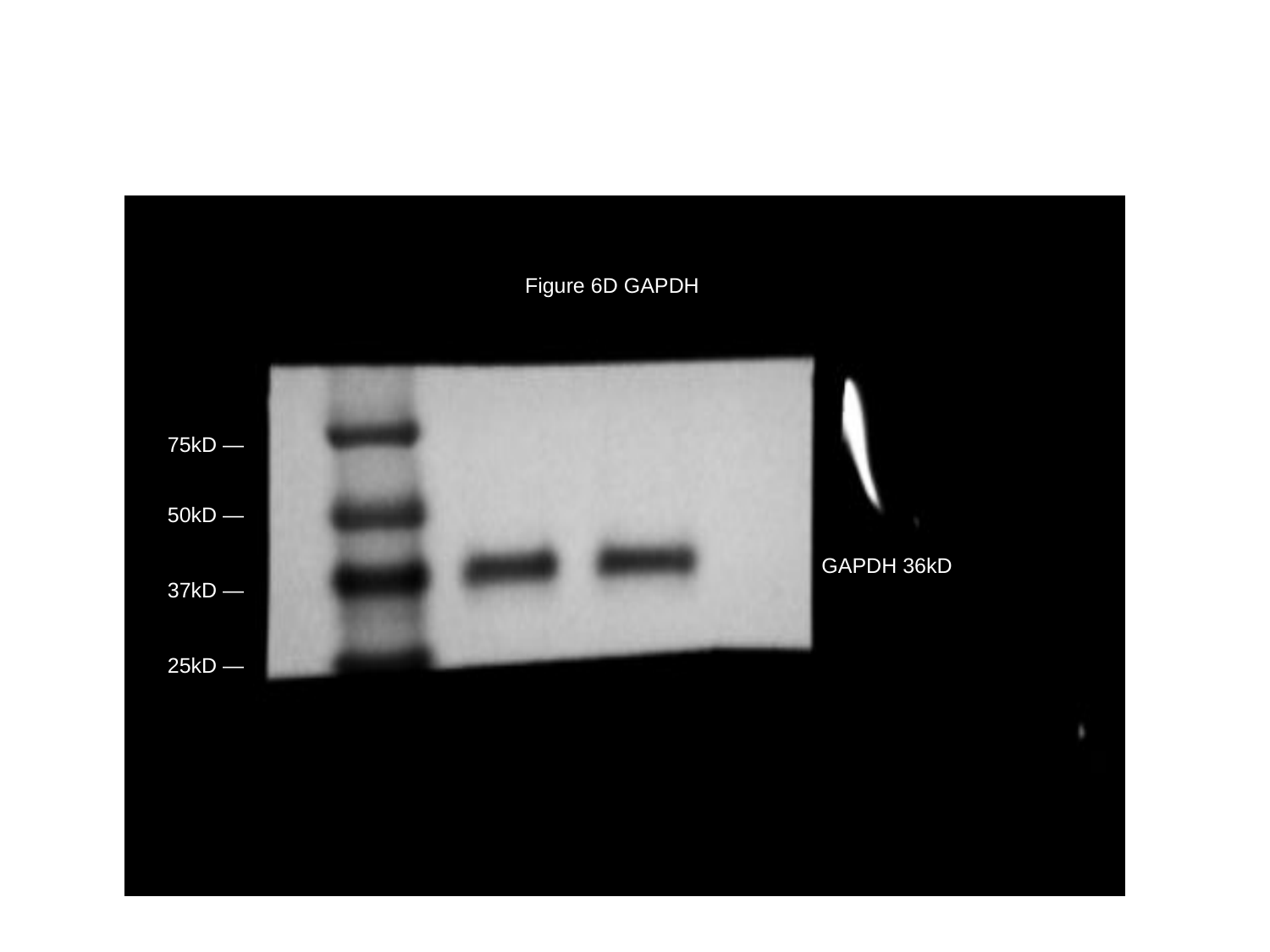

Figure 6D GAPDH
75kD —
50kD —
GAPDH 36kD
37kD —
25kD —

## Slide 16
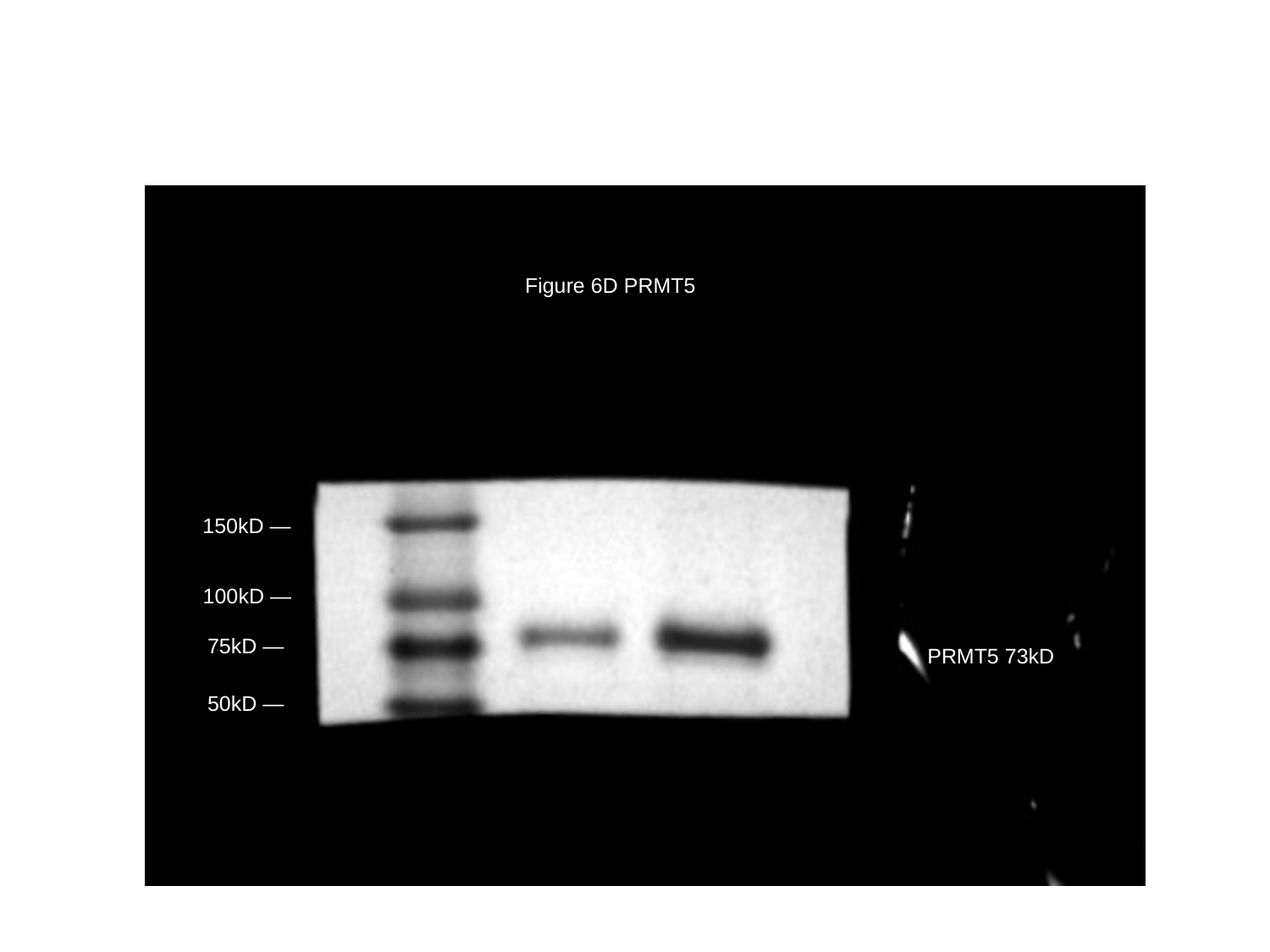

Figure 6D PRMT5
150kD —
100kD —
75kD —
PRMT5 73kD
50kD —

## Slide 17
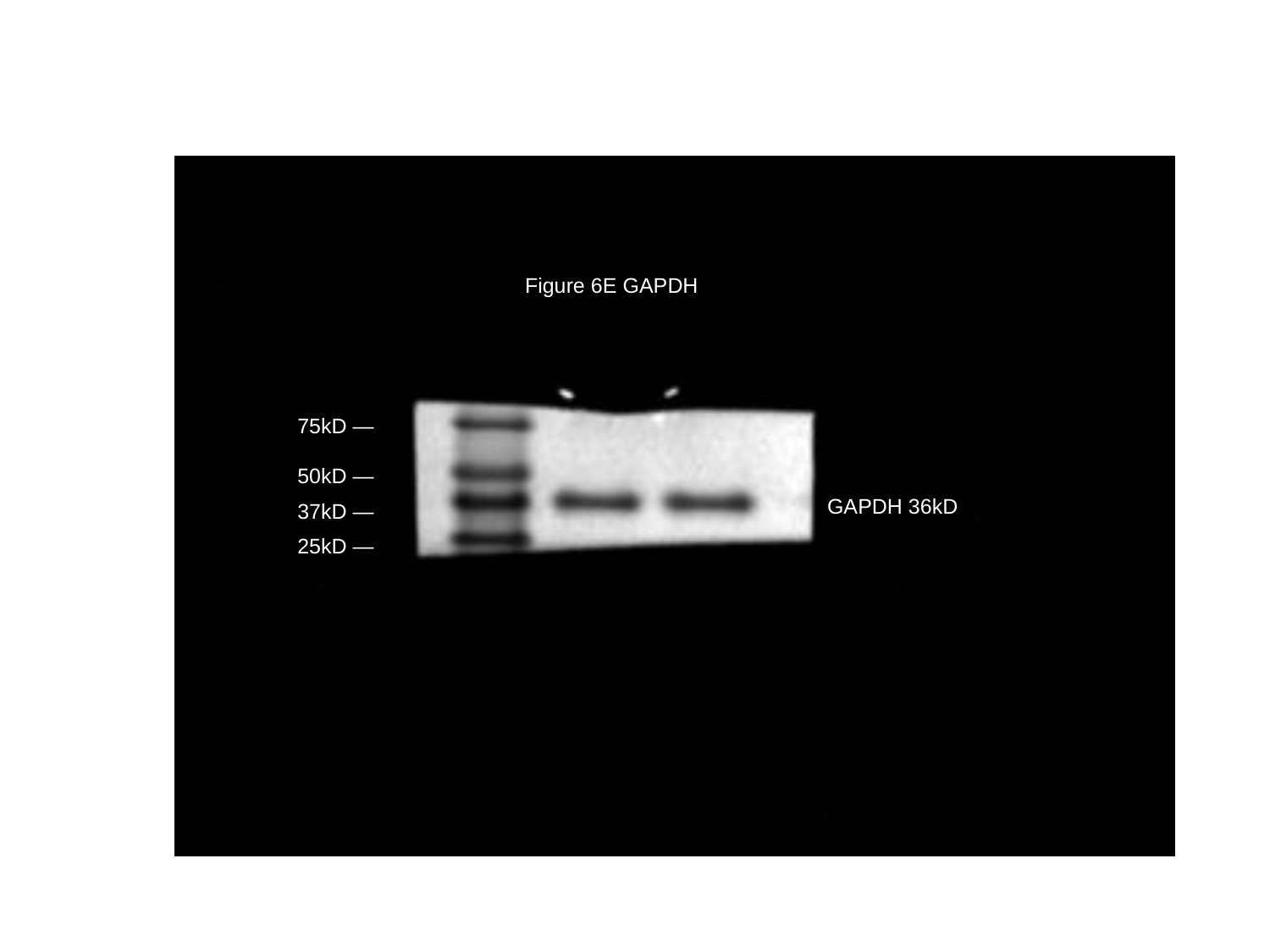

Figure 6E GAPDH
75kD —
50kD —
GAPDH 36kD
37kD —
25kD —

## Slide 18
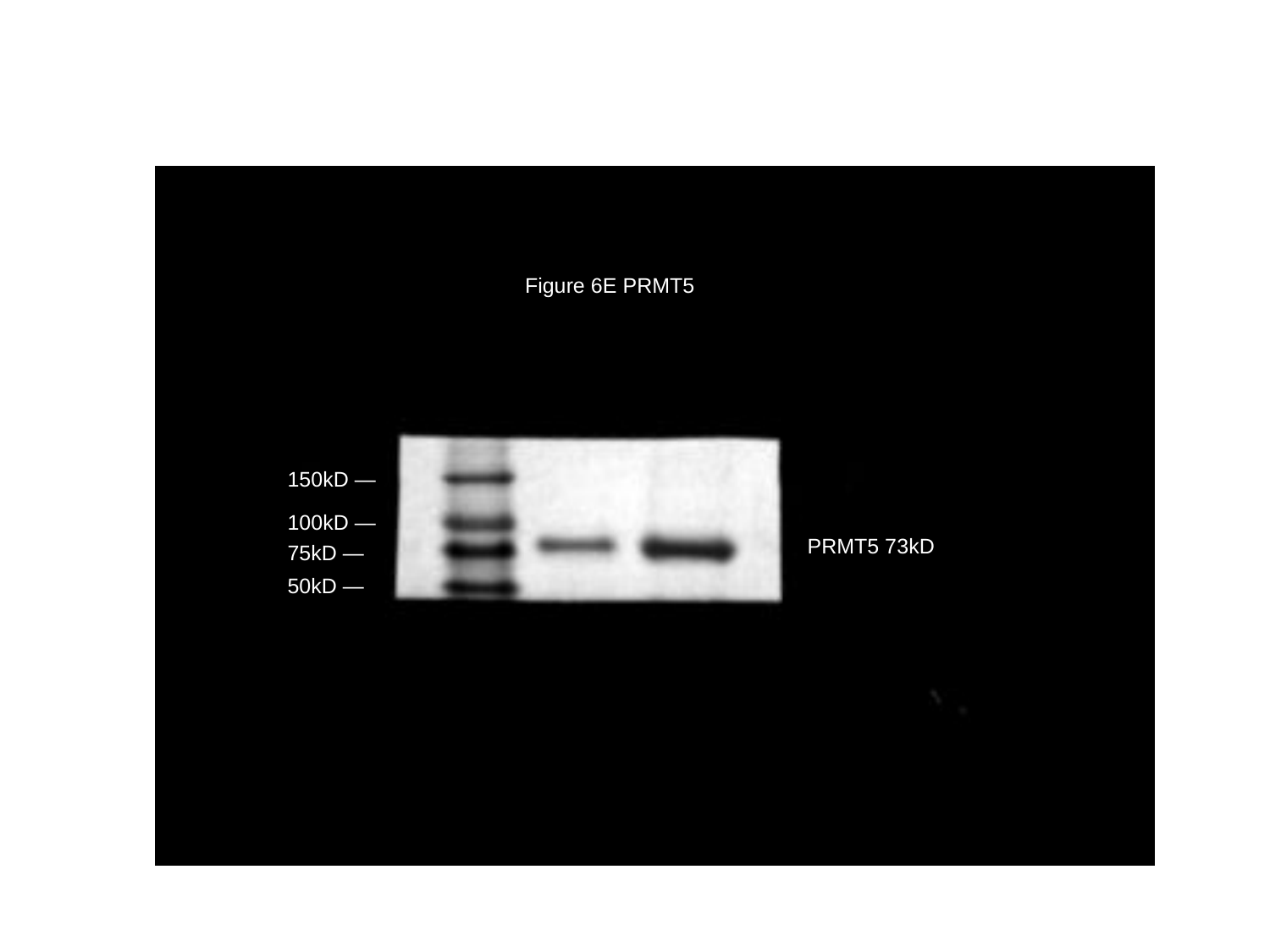

Figure 6E PRMT5
150kD —
100kD —
PRMT5 73kD
75kD —
50kD —

## Slide 19
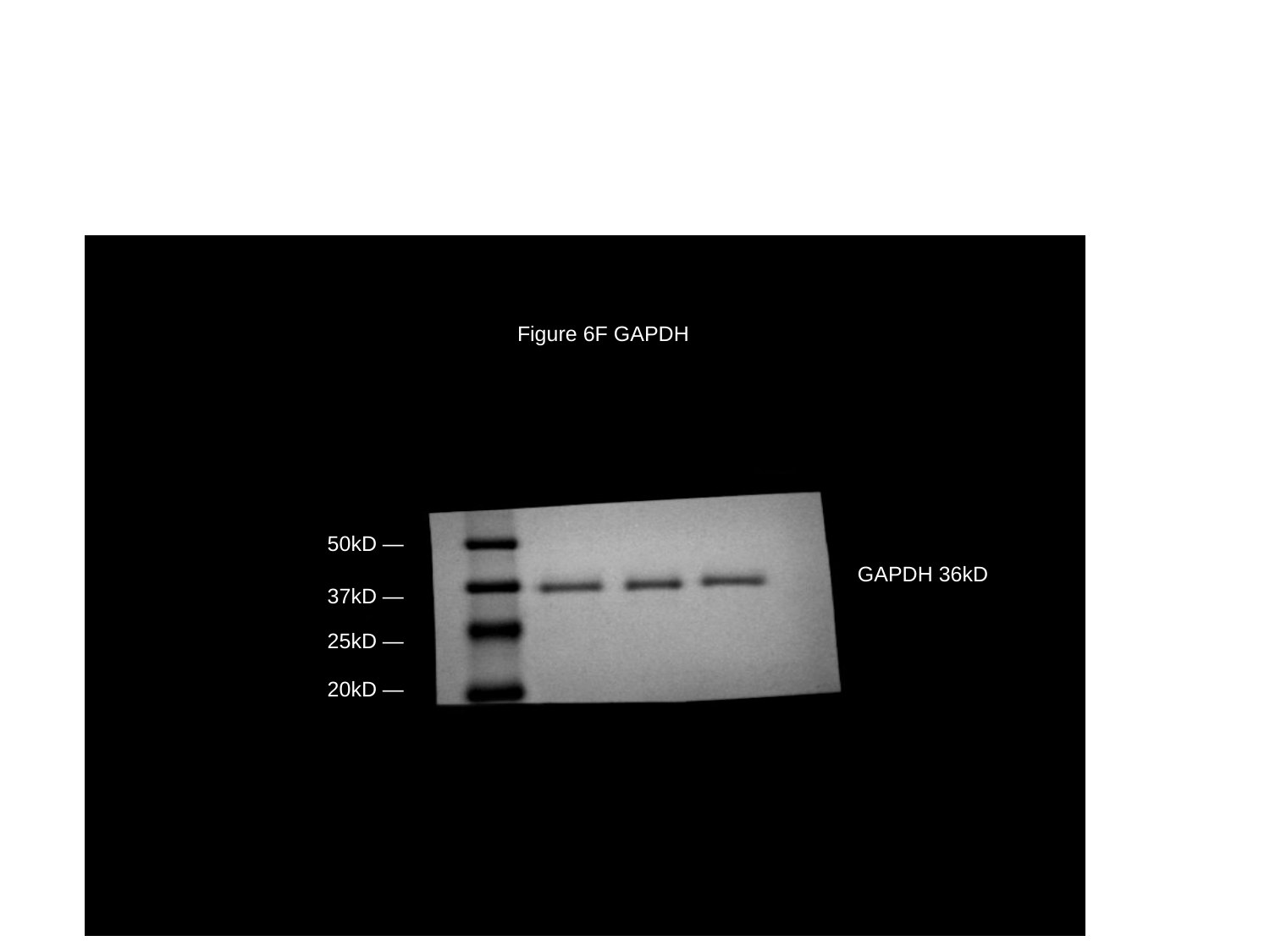

Figure 6F GAPDH
50kD —
GAPDH 36kD
37kD —
25kD —
20kD —

## Slide 20
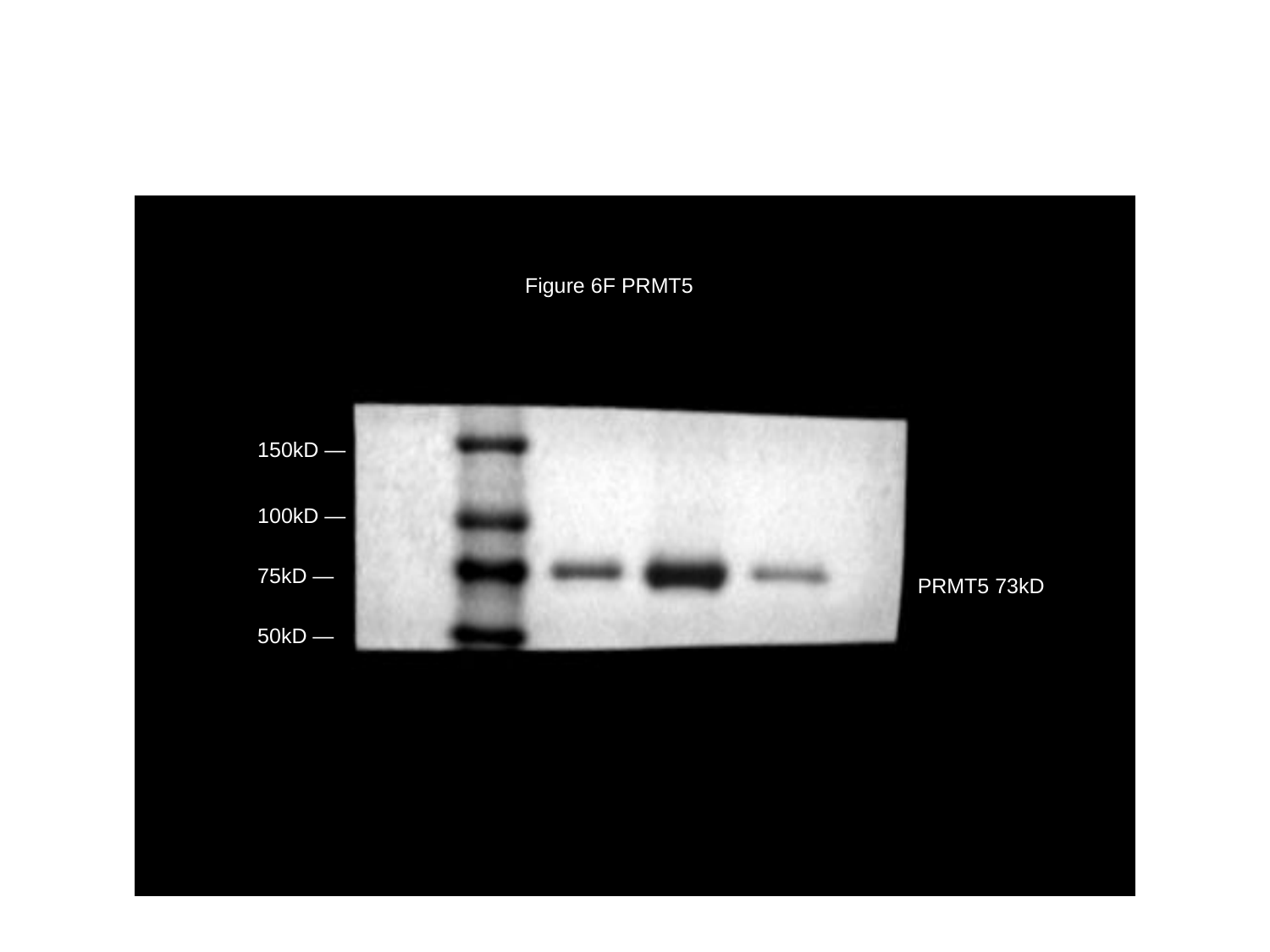

Figure 6F PRMT5
150kD —
100kD —
75kD —
PRMT5 73kD
50kD —

## Slide 21
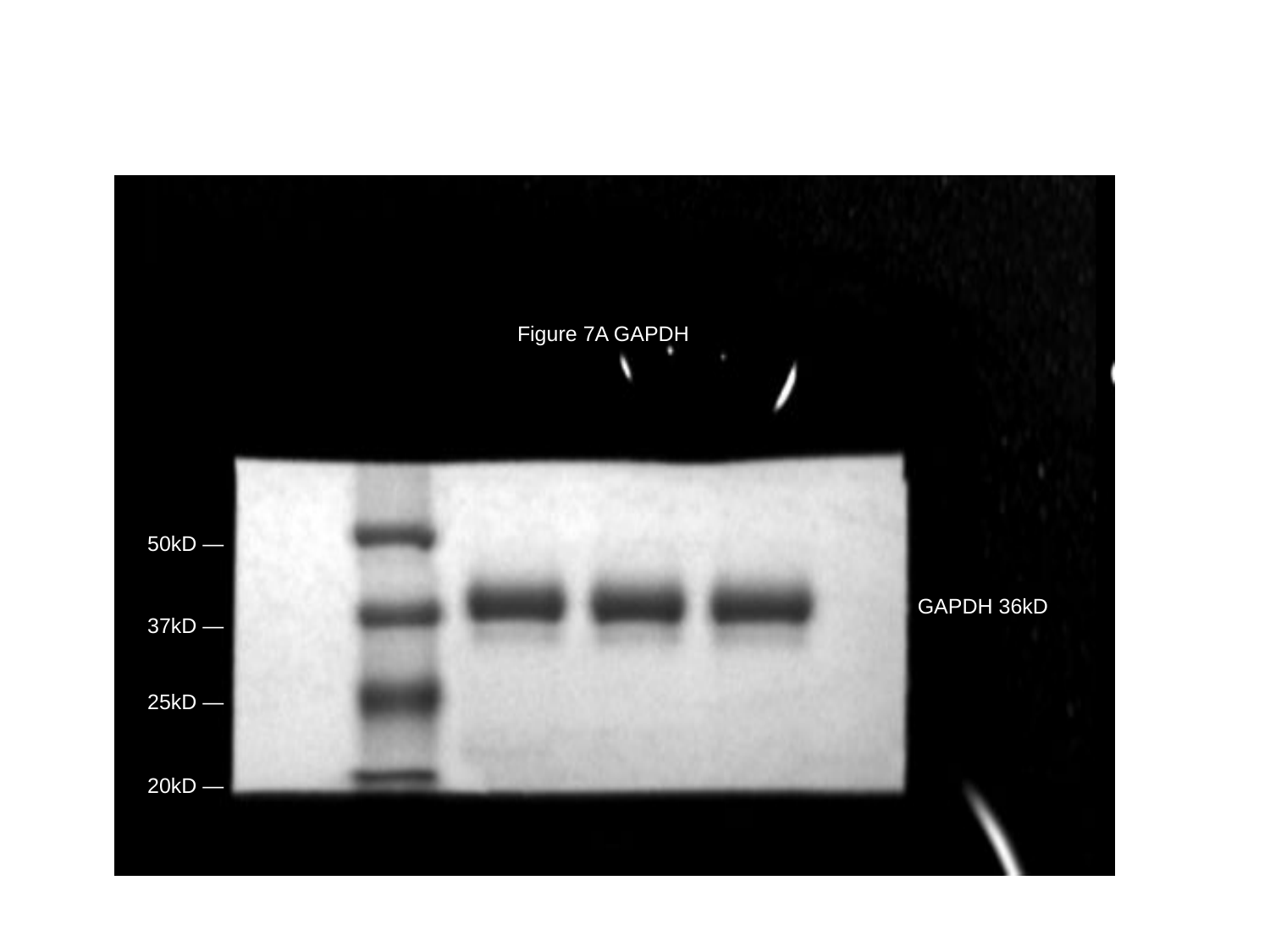

Figure 7A GAPDH
50kD —
GAPDH 36kD
37kD —
25kD —
20kD —

## Slide 22
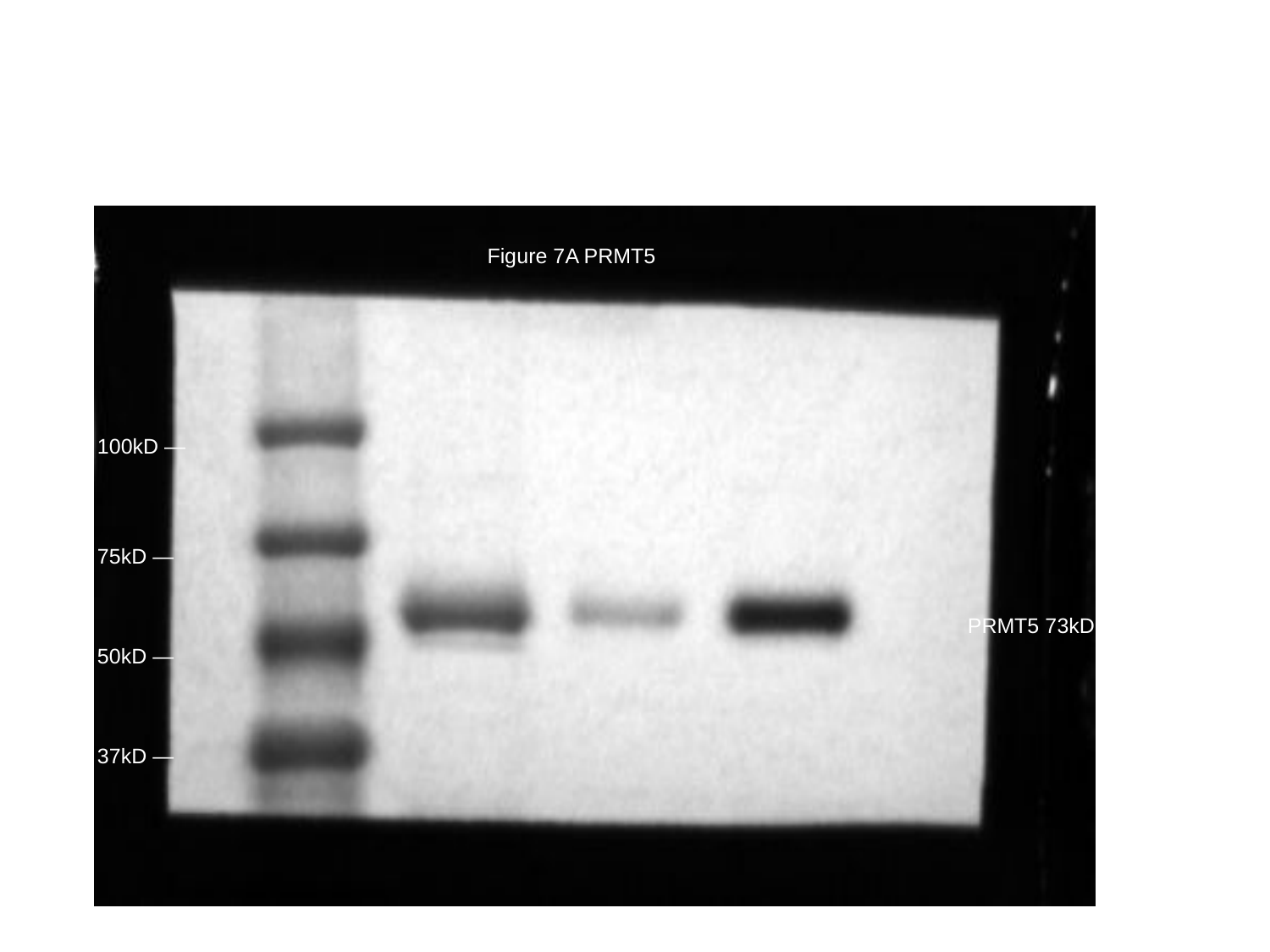

Figure 7A PRMT5
100kD —
75kD —
PRMT5 73kD
50kD —
37kD —

## Slide 23
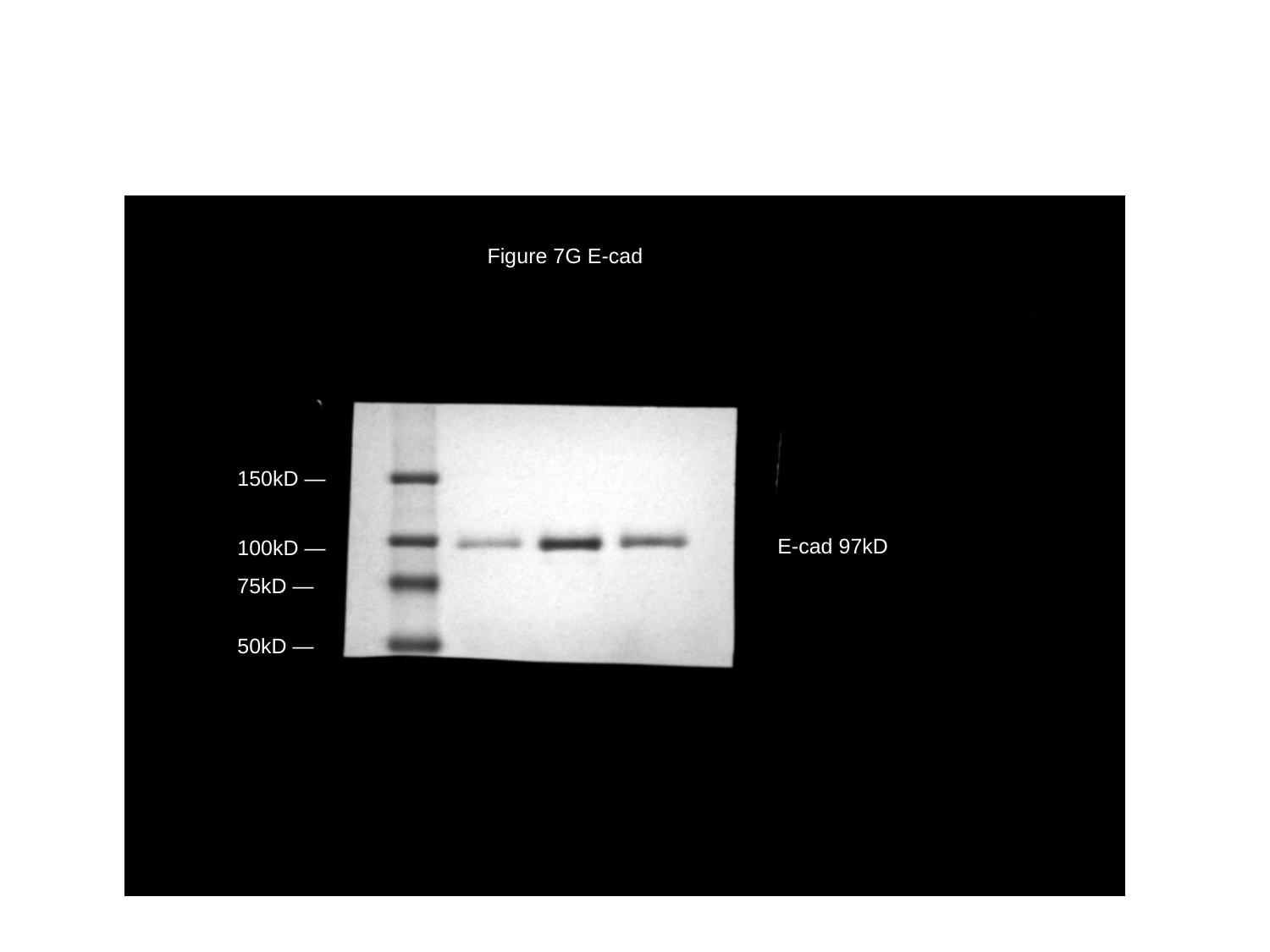

Figure 7G E-cad
150kD —
E-cad 97kD
100kD —
75kD —
50kD —

## Slide 24
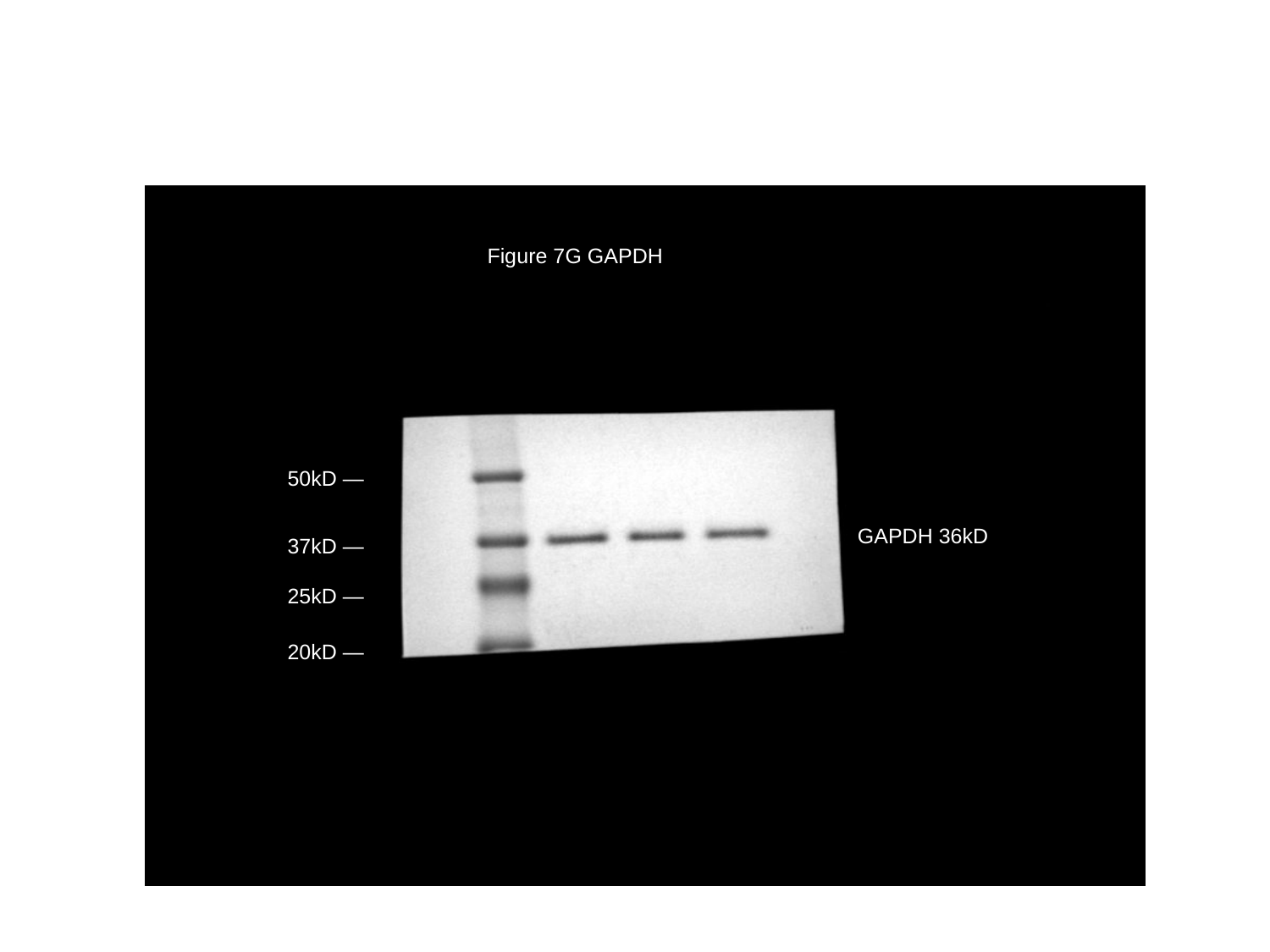

Figure 7G GAPDH
50kD —
GAPDH 36kD
37kD —
25kD —
20kD —

## Slide 25
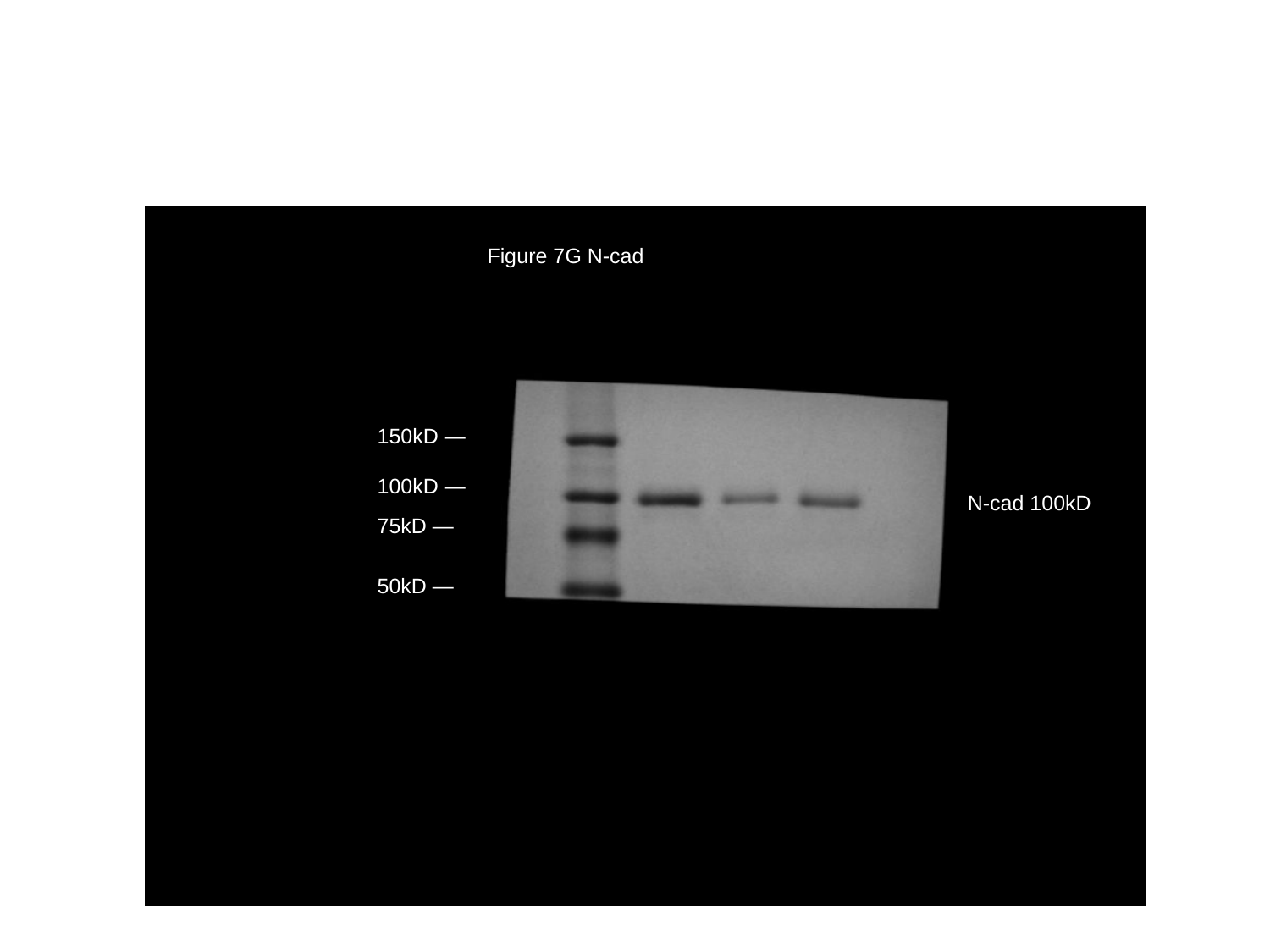

Figure 7G N-cad
150kD —
100kD —
N-cad 100kD
75kD —
50kD —

## Slide 26
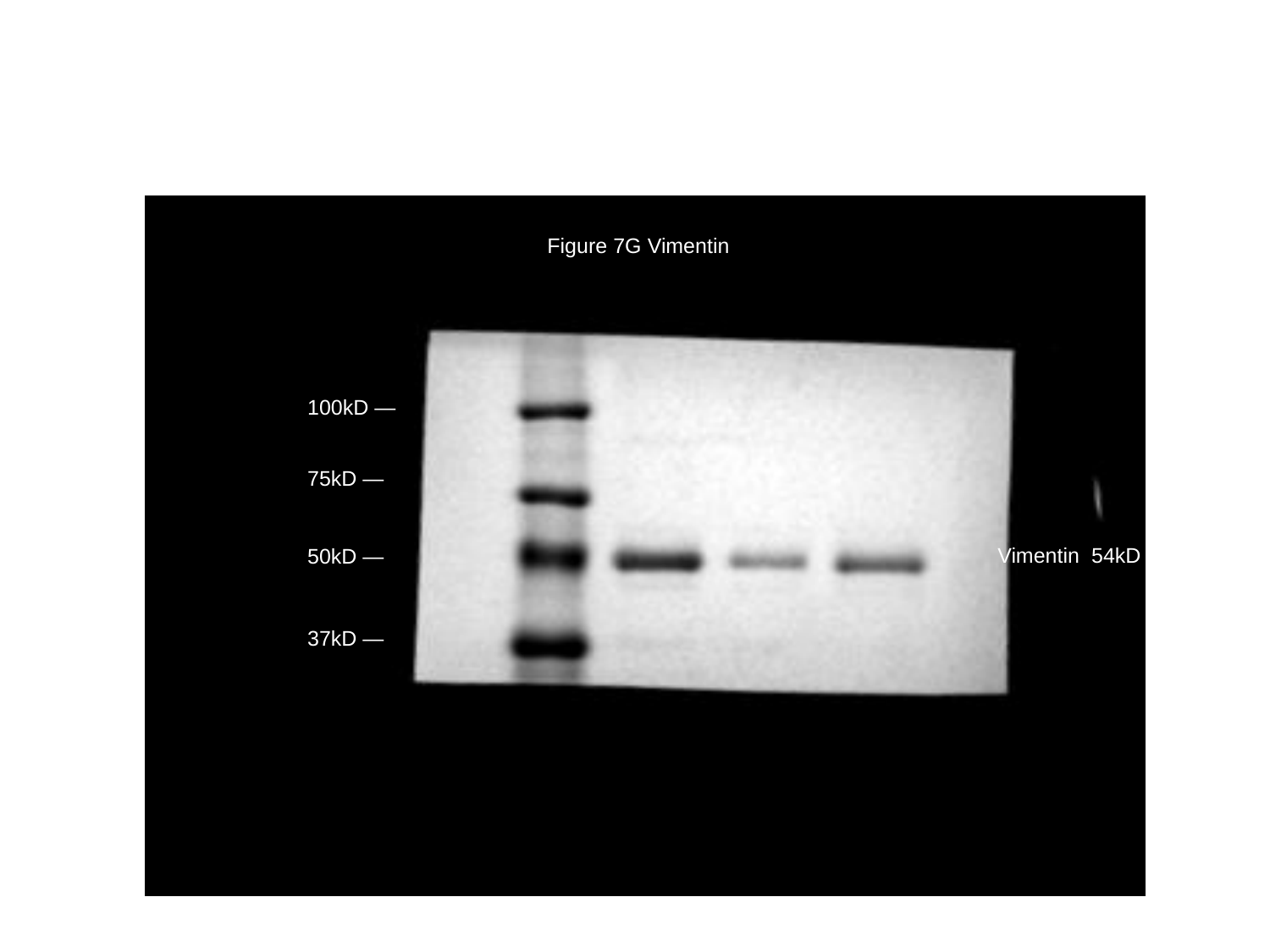

Figure 7G Vimentin
100kD —
75kD —
50kD —
Vimentin 54kD
37kD —

## Slide 27
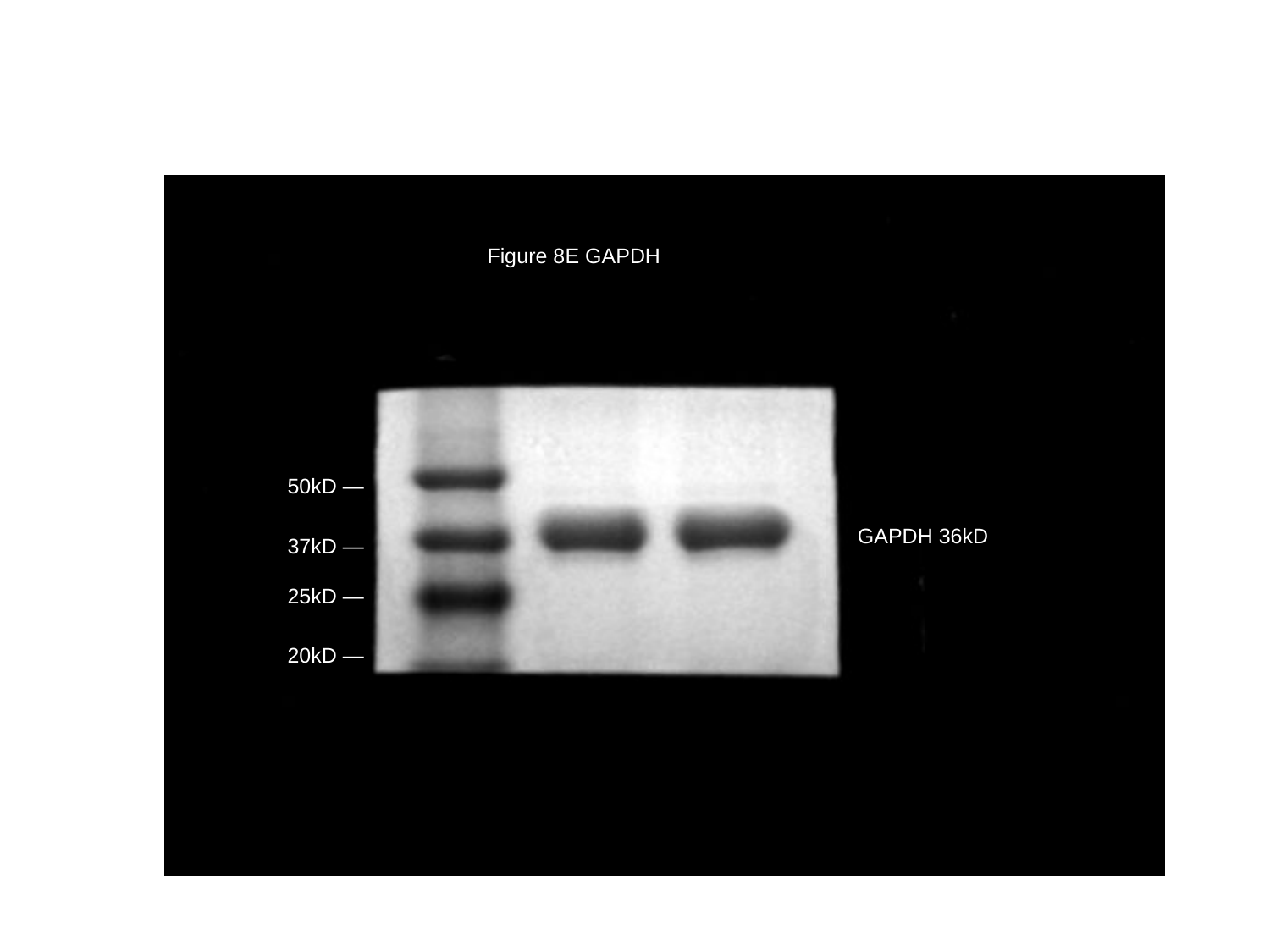

Figure 8E GAPDH
50kD —
GAPDH 36kD
37kD —
25kD —
20kD —

## Slide 28
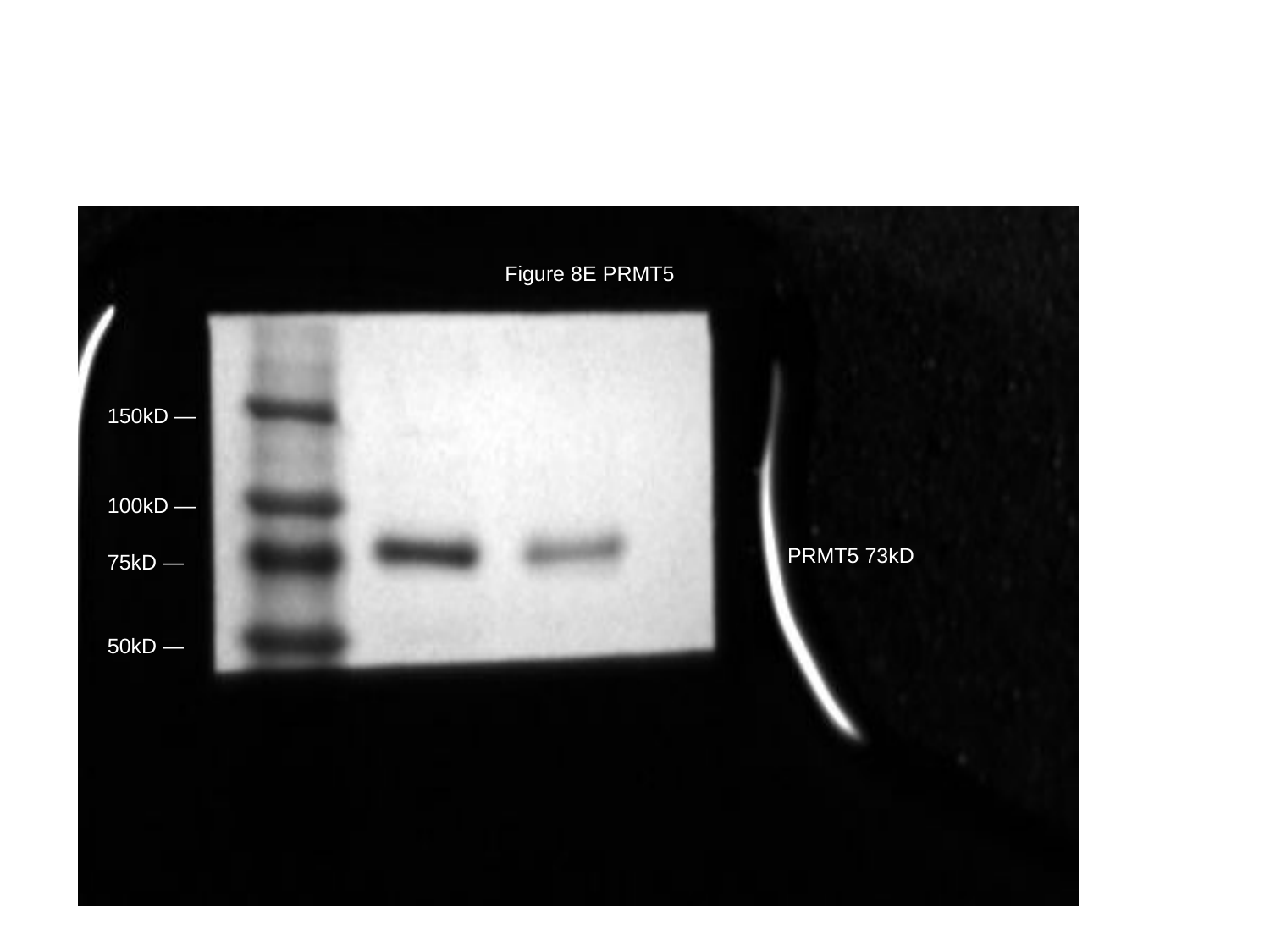

Figure 8E PRMT5
Figure 8E PRMT5
150kD —
100kD —
PRMT5 73kD
75kD —
PRMT5 73kD
50kD —
